# Chirality-Assisted Self-Assembly of Low-Symmetry Noncovalent Capsules with Quantitative Diastereoisomeric Selection

**DOI:** 10.1021/jacs.5c10523

**Published:** 2025-08-15

**Authors:** Grzegorz Markiewicz, Xujun Qiu, Gokay Avci, Emma H. Wolpert, Kim E. Jelfs, Jeremy K. M. Sanders, Artur R. Stefankiewicz

**Affiliations:** † Center for Advanced Technologies, 49562 Adam Mickiewicz University, Uniwersytetu Poznańskiego 10, 61-614 Poznań, Poland; ‡ Faculty of Chemistry, 49562 Adam Mickiewicz University, Uniwersytetu Poznańskiego 8, 61-614 Poznań, Poland; § Department of Chemistry, 4615 Imperial College London Molecular Science Research Hub, White City Campus, Wood Lane, London W12 0BZ, U.K.; ∥ Yusuf Hamied Department of Chemistry, 2152 University of Cambridge, Lensfield Road, Cambridge CB2 1EW, U.K.

## Abstract

One of the main aspects in which artificial capsules and cages still differ from their biological counterparts is symmetry—a fundamental trait that gives natural systems exceptional selectivity in molecular recognition. While the symmetry challenge has been recently addressed within metallosupramolecular or covalent systems, the creation of purely noncovalent capsular assemblies with tunable symmetry remains elusive. One exciting avenue toward reducing symmetry is to avail chirality in chiral-sensitive self-assembly, where symmetry is altered upon component binding. Herein, we report new pathways for the self-assembly of purely noncovalent capsules, wherein both the assembly outcome and symmetry can be finely adjusted through the strategic design of hydrogen bonding motifs and chirality. Specifically, depending on the mixture composition and hydrogen bonding centers embedded on the amino acid-derived benzene-1,3,5-triamide components, the system yields purely noncovalent capsules, ranging from highly symmetrical dimers to reduced symmetry octamers and new heterochiral tetrameric assemblies of low symmetry. The self-assembly processes presented here can be selectively modulated between narcissistic self-sorting pathways and diastereoselective social mixing, as well as toward the creation of social heterocomponent libraries—all yielding distinct capsular products. By showcasing the unique ability for molecular recognition, we believe our approach will pave the way for creating new capsular-type assemblies with unique functionalities and provide a fresh perspective on intricate, nature-inspired molecular dynamics.

## Graphical abstract

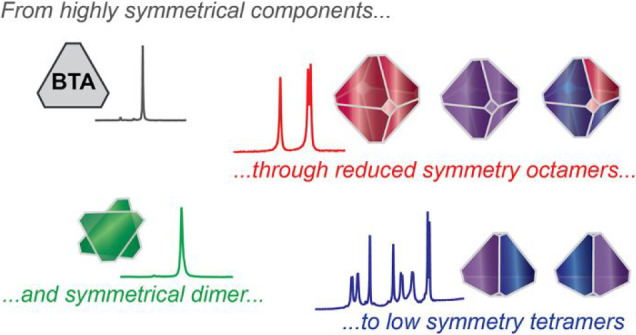

## Introduction

Noncovalent interactions are vital for all living organisms. Among these interactions, hydrogen bonds play a central role in biology, with their complex arrays being responsible for protein folding, enzyme activity, and DNA double helix formation.^1^ Inspired by nature,^2^ supramolecular chemists have explored multiple pathways for generating sophisticated hydrogen-bonded assemblies,^3^ tuning their properties^4^ and functionality through chemical design,^5^ environmental factors,^6^ or external stimuli.^7^ One extensively investigated group of these assemblies are supramolecular capsules,^8^ which have the ability to bind guest molecules within well-defined voids.^9^ This guest binding capability gives supramolecular capsules great potential in molecular sensing,^10,11^ molecular transport,^12,13^ or selective catalysis.^14–19^

One area where artificial systems still fall behind their biological archetypes is symmetry.^20^ Enzymes and their active sites possess inherently low symmetry, providing binding selectivity and functionality that remain unmatched by artificial systems.^21^ As a result, research has shifted toward methods of selective symmetry reduction,^20^ aiming to bypass narcissistic sorting or the formation of statistical mixtures. For example, metallosupramolecular chemists^22^ have tackled this issue using integrative self-sorting,^23^ where symmetry loss is achieved by accommodating different ligands within a cage structure,^24–26^ or through orientational self-sorting, where the isomeric selectivity is achieved via geometric constraints around the metal centers.^27–29^ Another approach involves the laborious synthesis of covalently bound organic cages or macrocycles, where desymmetrization is accomplished through stepwise organic transformations.^30–35^ Omitting the stepwise processes, Dynamic Covalent Chemistry (DCC) offers an alternative. For example, a figure-of-eight meso-knot is formed through the social mixing of enantiomers that individually form homochiral knots,^36^ or pseudopeptide heterocomponent cages can be isolated from dynamic covalent libraries (DCLs).^37^

However, reducing symmetry in purely noncovalent systems presents a unique challenge due to the inherently low interaction energies and the highly dynamic, elusive nature of these assemblies.^38^ Without convenient methods for eliminating side products or mismatched intermediates, researchers must rely on quantitative equilibria—common in nature, but rarely seen in artificial systems. As a result, it is unsurprising that the vast majority of reported hydrogen-bonded capsules are dimeric species, assembled from preformed cavitand-type molecules, often stable only in the presence of a template molecule.^8,39–43^

Over the years, only a handful of motifs have been developed that form multimembered noncovalent capsules, ranging from tetrameric,^45^ hexameric,^46^ octameric,^47^ decameric,^48^ to anion-sealed dodecameric species.^49,50^

Herein, we present an independent approach toward low-symmetry capsules that form spontaneously from highly symmetrical noncavitand building blocks based on a benzene-1,3,5-tricarboxylic acid (BTA) core. With the aid of chirality and precisely designed hydrogen bonding arrays around the BTA core, we not only shifted the assembly pathway from the most common columnar-type polymers^51^ to capsules,^44^ but also accomplished their structural and symmetry reorganization on demand. Depending on the mixture’s composition, the system yields purely noncovalent capsules with different structures, ranging from highly symmetrical dimers and reduced symmetry octamers, to novel heterochiral tetrameric assemblies of low symmetry. Demonstrating its unique capability for molecular and chirality recognition, the supramolecular system exhibits tendencies toward narcissistic sorting of capsules, diastereoselective social mixing into well-defined assemblies, and the creation of social heterocomponent libraries—all governed by complex hydrogen-bonded equilibria (Figure 1).

**Figure 1:**
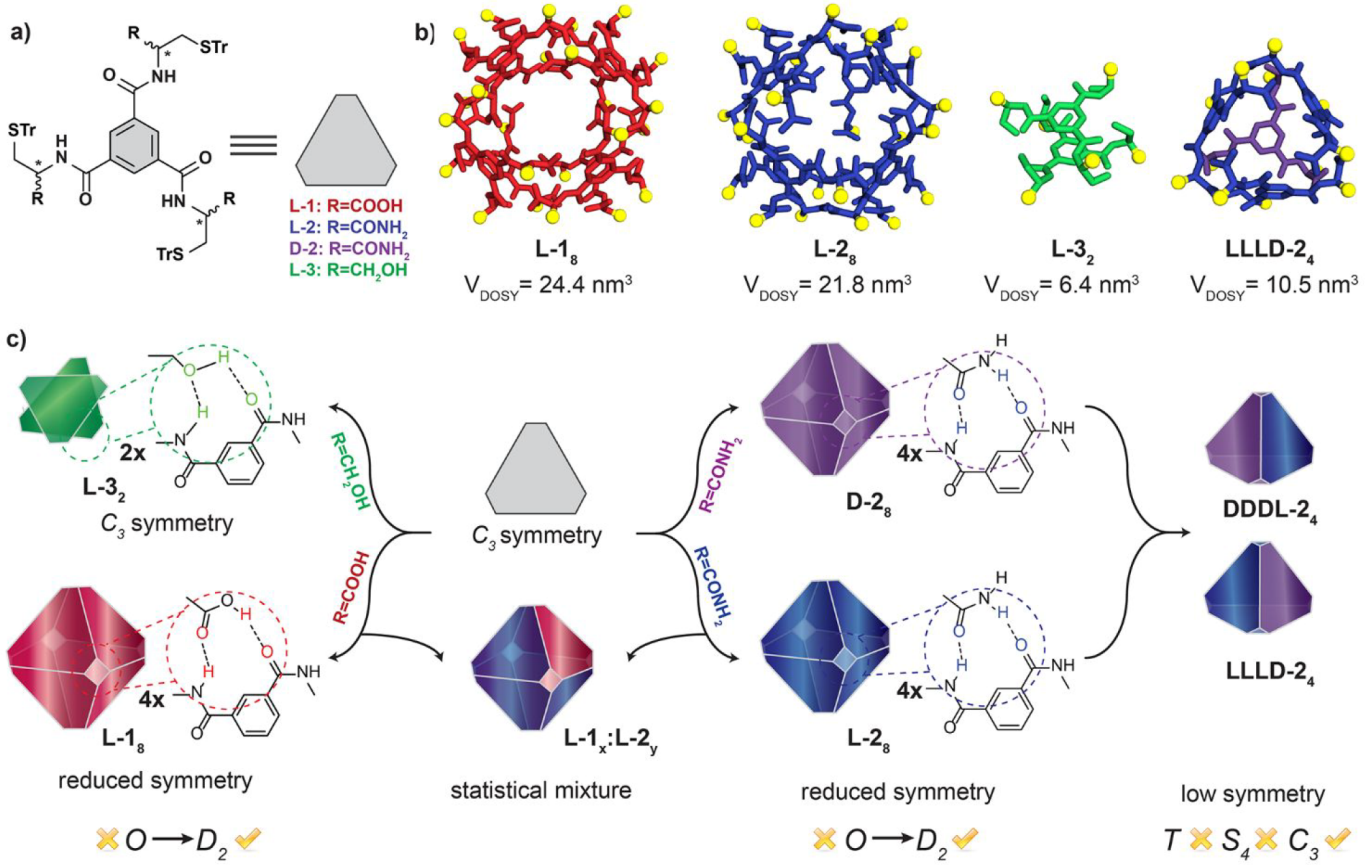
(a) Chemical structures of l-**1**, l-**2**, d-**2** , l-**3**. (b) Ball and stick models showing: X-ray structure of l-**1**_8_ (red),^44^ computational model of l-**2**_8_ (blue), computational model of l-**3**_2_ (green), computational model of llld-**2**_4_ (blue/violet) with solvodynamic volumes determined from ^1^H NMR DOSY experiments in TCE-*d*_2_. For clarity, *S*(Tr) units were replaced with methyls (yellow balls), and hydrogen atoms are omitted from visualization. (c) Schematic representation of the self-assembly outcomes of l-**1**, l-**2**, l-**3** components as observed in TCE solutions.

## Results and Discussion

The building blocks l-**2** and d-**2** (Figure 1a) were synthesized by reacting enantiopure *S*(Tr)-Cys-amide with an activated ester of BTA, following the previously reported procedure for l-**1/** d-**1**.^44^ Building block l-**3** (Figure 1a) was synthesized in two steps, starting from component l-**1**, which was esterified with methanol to afford its methyl ester, followed by the reduction of the ester group to an alcohol with LiBH_4_ (see Section 1.2 in Supporting Information for details).

### Homochiral Assemblies

The newly synthesized building blocks l-**2** and l-**3** were first studied by ^1^H NMR spectroscopy in different solvents—dimethyl sulfoxide (DMSO-*d*_6_) and 1,1,2,2-tetrachloroethane (TCE-*d*_2_)—and compared to the previously reported component l-**1** (Figure 2). In the highly polar DMSO-*d*_6_, the ^1^H NMR spectra of all three building blocks showed a set of sharp and well-resolved peaks corresponding to the solvated monomers, with *C*_3_-symmetry, indicating that any intermolecular H-bonding between monomers was blocked by interactions with the solvent. However, in the apolar TCE-*d*_2_, the ^1^H NMR spectra were markedly different, with broadened and shifted peaks compared to those in DMSO-*d*_6_, indicating the formation of presumably H-bonded aggregates. In the case of l-**2** (Figure 2d), proton signals for NH, NH_2_, and the α-proton were shifted upfield and broadened significantly, unambiguously indicating the formation of hydrogen-bonded aggregates. Additionally, the number of the peaks for each proton was tripled when compared to the spectrum in DMSO-*d*_6_ (Figure 2c) reflecting a decrease in symmetry from *O* down to *D*_2_ (432 to 222 after Hermann–Mauguin).^44^ This is reminiscent of the octameric hydrogen-bonded capsule l-**1**_8_,^44^ suggesting that l-**2** also self-assembles into l-**2**_8_ (Figure 1b). Notably, although both octamers appear to form octahedra, the complex hydrogen bonding arrays within those assemblies (vide infra) lead to a deformation of platonic polyhedrons, resulting in symmetry reduction.^44^

**Figure 2:**
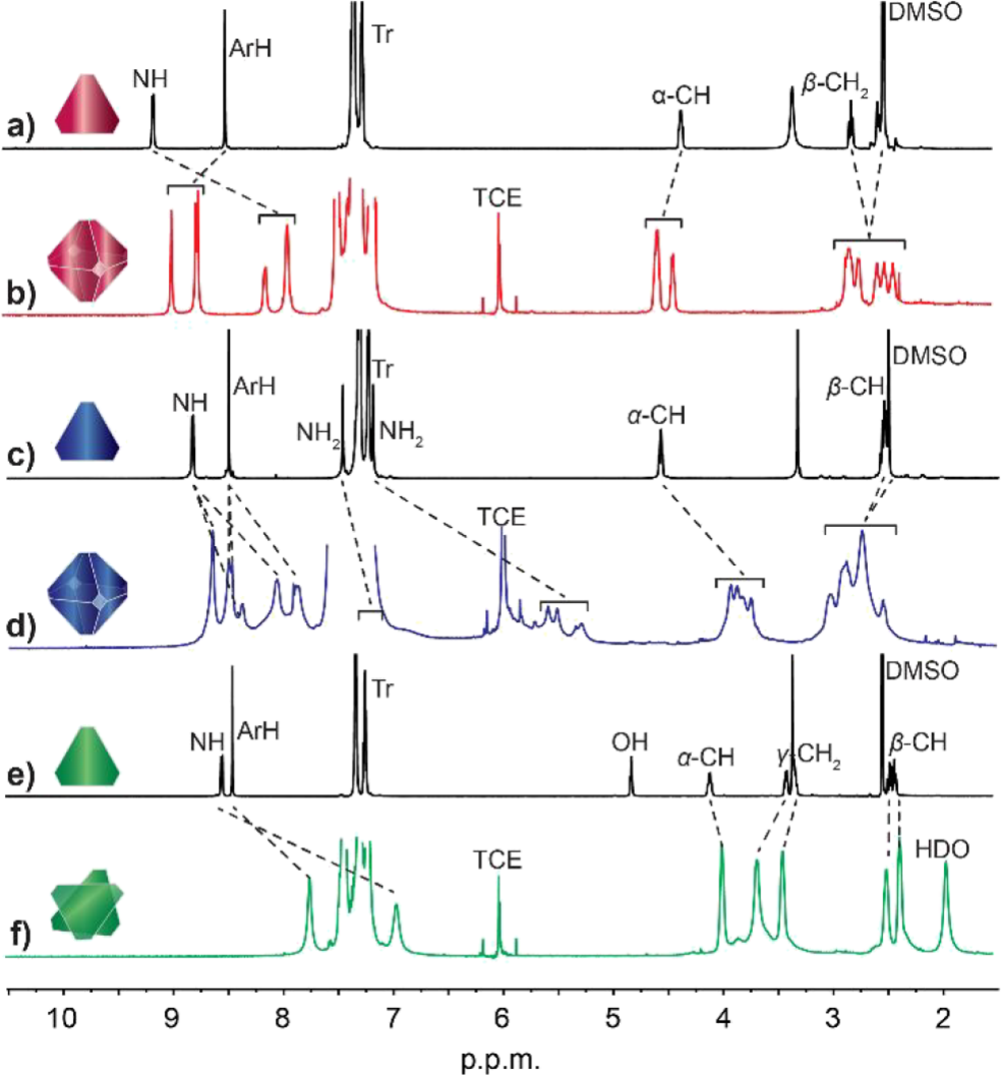
^1^H NMR (600 MHz) spectra of: (a) l-**1** in DMSO-*d*_6_; (b) l-**1** in TCE-*d*_2_; (c) l-**2** in DMSO-*d*_6_; (d) l-**2** in TCE-*d*_2_; (e) l-**3** in DMSO-*d*_6_; (f) l-**3** in TCE-*d*_2_. Insets show the assembly states observed in solution.

For l-**3**, the peaks were broadened and shifted upfield, i.e., Ar CH from 8.4 to 7.7 ppm, NH from 8.5 to 6.9 ppm, α-proton from 4.0 to 3.9 ppm (Figure 2f). However, no desymmetrization was observed during the self-assembly of l-**3**, suggesting that the self-assembled supramolecular aggregate retained the *C*_3_ symmetry of a monomer. The simplest species for which this is possible is the dimer l-**3**_2_.

A change in the assembly outcome from an octameric to a dimeric product suggests that their sizes should differ significantly. The latter was investigated using diffusion-ordered NMR spectroscopy (DOSY NMR) to estimate the solvodynamic radii of the assemblies. For the octameric capsule l-**1**_8_, the hydrodynamic radius was estimated to be 18.2 Å,^44^ while a nonaggregated monomer had a radius of 10.6 Å.^44^ Using the Stokes–Einstein equation, the solvodynamic radii of the anticipated octameric (l-**2**_8_) and dimeric (l-**3**_2_) capsules in TCE-*d*_2_ were found to be 17.3 Å with a diffusion coefficient 8.62 × 10^–11^ m^2^ s^–1^ (Figure S14 in Supporting Information) and 11.1 Å with a diffusion coefficient of 1.35 × 10^–10^ m^2^ s^–1^ for l-**3**_2_ (Figure S15 in Supporting Information), respectively. These values align well with the expected sizes of the assembled structures. Following this, FT-IR (Figure 3a) analysis of l-**3** recorded in TCE (for spectra in DMSO and THF see Figure S9 in Supporting Information) revealed broadened ν­(*N–H*)_*amide*_ and ν­(*O–H*)_*alcohol*_ bands at 3410 and 3350 cm^–1^, and ν­(*C=O*)_*amide*_ at 1665 cm^–1^—all consistent with the formation of an H-bonded assembly.^52–54^

**Figure 3:**
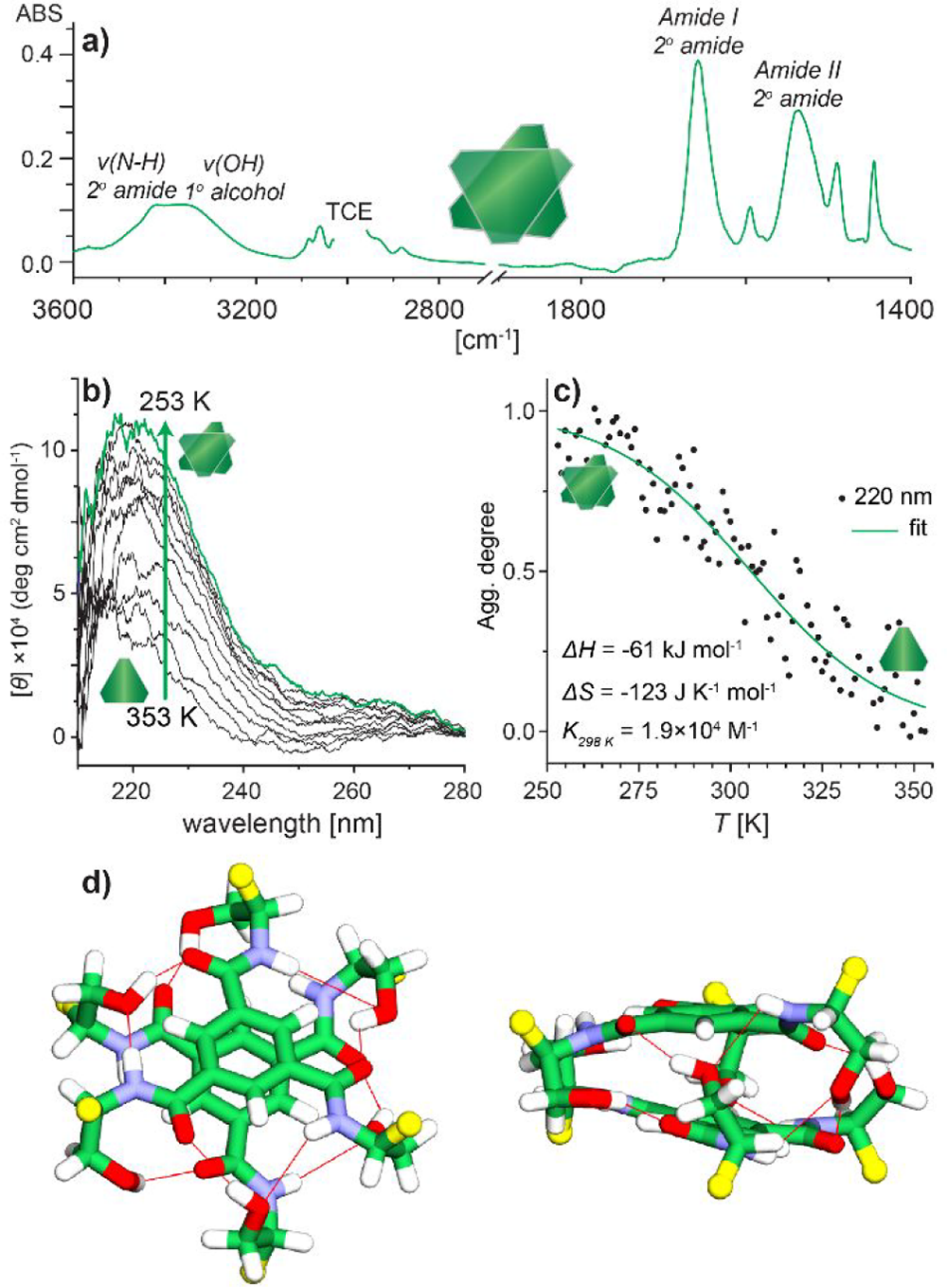
(a) Partial FT-IR spectrum of l-**3**_2_ in TCE solution (*C* = 1.0 × 10^–2^ m). (b) Variable temperature (VT) CD spectra recorded during cooling of a solution of l-**3** in DCE (*C* = 1.0 × 10^–4^ m, *T* = 353–253 K, cooling rate: −1 K min^–1^). (c) Normalized temperature-dependent CD intensity recorded during cooling of a solution of l-**3** (*C* = 1.0 × 10^–4^ m, *T* = 353–253 K, cooling rate: −1 K min^–1^). (d) Computational model of l-**3**_2_ with marked hydrogen bonding (red lines). For clarity, *S*(Tr) units were replaced with methyls (shown here as yellow balls), and hydrogen atoms are omitted from visualization.

The formation of H-bonded/π-stacked dimers is commonly observed for the BTA derivatives,^3,51^ and in the case of chiral molecules (like l-**3**) is also manifested by changes in supramolecular chirality.^3,51^ Indeed, the circular dichroism (CD) spectrum of l-**3** in 1,2-dichloroethane (DCE) (Figure 3b) shows a very marked temperature dependence, indicating that it could be used as a quantitative probe of the anticipated dimerization equilibrium. Notably, TCE had to be replaced with DCE in this experiment since TCE absorbs UV light below 270 nm. The changes in ellipticity at λ = 220 nm (Figure 3c) observed upon cooling were sigmoidal—a transition characteristic of either dimerization or an *equal-K* isodesmic polymerization process. Although both processes are indistinguishable from the fitting,^55^ the apparent DOSY size and steric bulkiness of trityl units leaves dimerization as the only possible outcome of the assembly.

The thus obtained VT-CD data have been analyzed via van’t Hoff relations, using the methodology previously utilized by us for the H-bonded dimeric capsule,^56^ and revealed an enthalpy change of Δ*H* = −61 kJ mol^–1^, entropy change of Δ*S* = −123 J K^–1^ mol^–1^ and the dimerization equilibrium constant of *K*_298 K_ = 1.9 × 10^4^ m ^–1^. The values are consistent with the enthalpy-driven formation of a dimeric assembly held together by noncovalent interactions.

Computational modeling for the l-**3**_2_ dimer (Figure 3d; see Section 2 and Figure S35 in Supporting Information for details) shows that it forms 12 hydrogen bonds between the OH^···^O=C and NH^···^O groups, or 6 per molecule, which is fewer than the 8 hydrogen bonds per molecule formed for the octamer capsules. This is due to the absence of a carbonyl group’s oxygen atom in l-**3**, which contributes to hydrogen bonding in the octameric capsule.

The DOSY analysis indicates that the amide-based l-**2**_8_ capsule is slightly smaller than the l-**1**_8_ capsule assembled from the carboxylic acid analog. This difference indicates a shrinking of the capsule, presumably due to a tighter hydrogen bonding array within the capsule core. To investigate this further, both octamers were characterized by FT-IR analysis in TCE solutions, allowing direct observation of the groups involved in hydrogen bonding formation (Figure 4a,b).^52^ The previously reported crystal structure of l-**1**_8_ (Figure 1b) revealed that the octameric assembly is held together by a complex array of 48 cooperative hydrogen bonds, distributed evenly around six pores located at the vertices of the capsule.^44^ Each pore is constructed by four pairs of OH^···^O=C and NH^···^O=C hydrogen bonds, with the acidic COOH group bridging two adjacent arms from another l-**1** molecule (Figure 1c).

**Figure 4:**
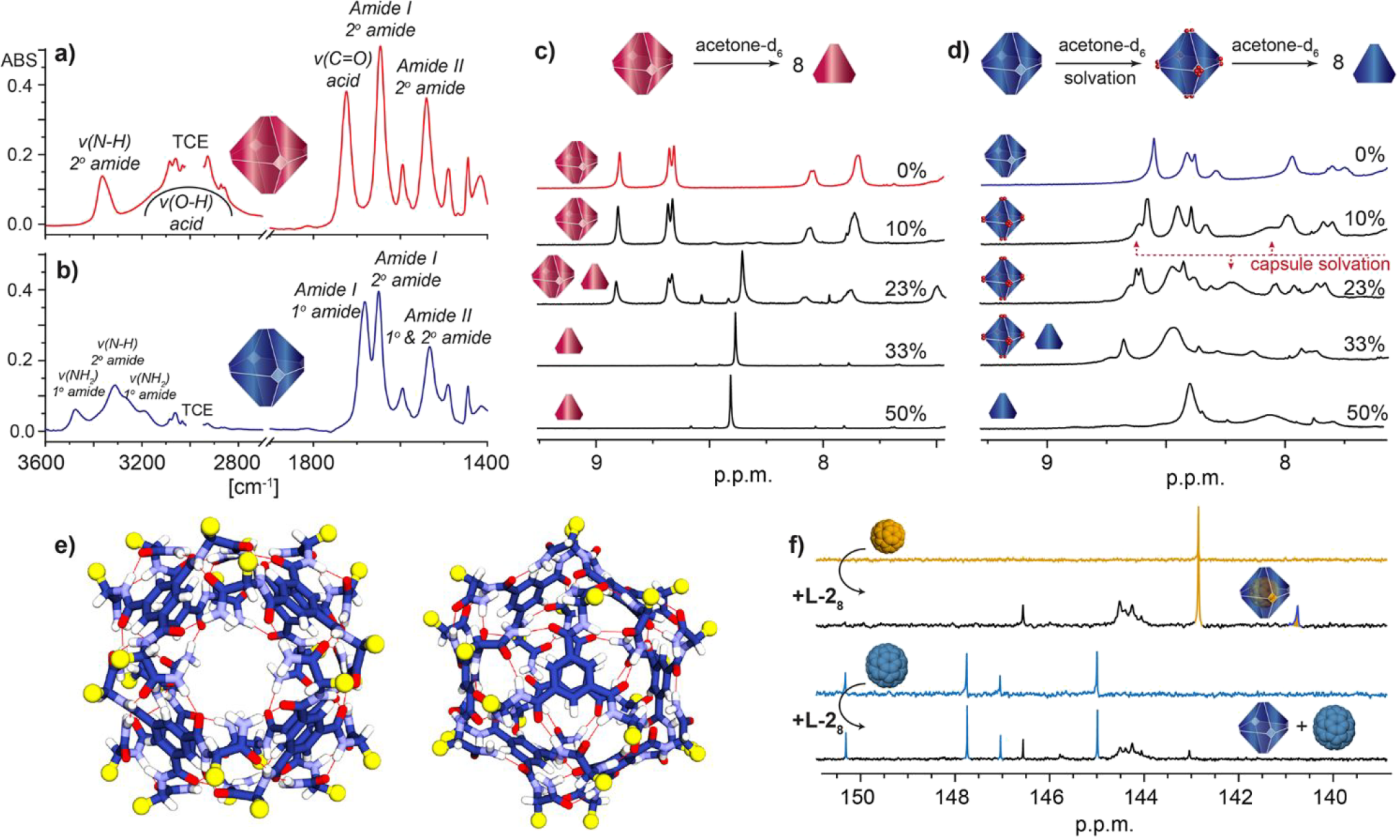
(a) Partial FT-IR spectrum of l-**1**_8_ in TCE solution (*C* = 1.0 × 10^–2^ m). (b) Partial FT-IR spectrum of l-**2**_8_ in TCE (*C* = 1.0 × 10^–2^ m). (c) Disassembly of the l-**1**_8_ capsule upon addition of acetone-*d*_6_, monitored by ^1^H NMR (600 MHz) in TCE-*d*_2_ solution (*C* = 1.0 × 10^–2^ m, inserts show the assembly states observed in solution); (d) Disassembly of the l-**2**_8_ capsule upon addition of acetone-*d*_6_ monitored by ^1^H NMR (600 MHz) in TCE-*d*_2_ solution (*C* = 1.0 × 10^–2^ m, insets show the capsule assemblies observed in solution). (e) Computational model of l-**2**_8_ with marked hydrogen bonding array (red lines). Views on the pore (left) and BTA wall (right) are presented. For clarity, *S*(Tr) units were replaced with methyls (yellow balls). (f) ^13^C NMR (151 MHz, TCE-*d*_2_) spectra of C_60_ and C_70_ fullerenes with and without l-**2**_8_ (insets show the assembly states observed in solution).

The FT-IR spectrum of l-**1**_8_ recorded in TCE (Figure 4a) was consistent with this array, showing broadened ν­(*N–H*)_*amide*_ and ν­(*O–H*)_*acid*_ bands at 3350 and 3000 cm^–1^, respectively. Both C=O groups were also found to be H-bonded, with ν­(*C=O*)_acid_ and ν­(*C=O*)_amide_ bands at 1720 and 1645 cm^–1^, respectively (for spectra in DMSO and THF see Figure S7 in Supporting Information). A closer look at the ν­(*N–H*)_*amide*_ band revealed two overlapping bands centered at 3360 and 3345 cm^–1^, indicating minor differences in H-bonding energy and geometry, which also contribute to breaking the symmetry of the capsule, as observed in the ^1^H NMR spectra.

A similar hydrogen bonding pattern was observed in the TCE solution of l-**2**_8_ (Figure 3b), where both the primary and secondary amide C=O groups participated in hydrogen bonding, as indicated by ν­(*C=O*) bands recorded at 1685 and 1650 cm^–1^, respectively (for spectra in DMSO and THF see Figure S8 in Supporting Information). The ν­(*N–H*)_*amide*_ band for the secondary amide (inner group) was observed at 3315 cm^–1^, indicating stronger H-bonds with the C=O acceptor compared to l-**1**_8_. The ν­(*N–H*)_*amide*_ bands for the primary amide (outer group) displayed a more complex pattern, with at least four bands centered at 3470, 3450, 3260, 3190 cm^–1^. Along with ^1^H NMR analysis, this supports the proposed hydrogen bonding pattern (Figure 1c), where only one of the hydrogen atoms from NH_2_ group is involved in hydrogen bonding.^52^ This observation was further supported by computational modeling (Figure 4e; see Section 2 and Figure S34 in Supporting Information for details). l-**2**_8_ forms a total of 48 hydrogen bonds per capsule, with the secondary amide acting as both a donor and acceptor, and the primary amide acting as a donor with the C=O acceptor, resulting in an extended hydrogen bonded network (Figure 4e).

To further evaluate the stability of the octameric assemblies, both capsules l-**1**_8_ and l-**2**_8_ in TCE-*d*_2_ solutions were titrated with hydrogen bonding competitive solvents, specifically acetone-*d*_6_ as a hydrogen bonding acceptor, and methanol-*d*_4_ as a competitive acceptor–donor (Figures 4c,d, and S7 in Supporting Information). We found that the l-**1**_8_ capsule remains stable up to 10% (v/v) of acetone-*d*_6_ in solution, after which a gradual disassembly of the octamer into solvated monomers was observed, with complete dissociation occurring above 25% acetone-*d*_6_ (Figure 4c). In contrast, the amide-based l-**2**_8_ capsule exhibits different behavior (Figure 4d). Initially, l-**2**_8_ appears to form H-bonds with the acetone-*d*_6_ molecules through its unoccupied NH centers, as evidenced by the strong deshielding of the free NH resonances by ≈3 ppm (from 5.5 to 8.5 ppm). This new complex remains stable up to 30% of acetone-*d*_6_, with complete disassembly into solvated monomers occurring at 50% of acetone-*d*_6_ in TCE-*d*_2_ solution. A similar difference in hydrogen bonding stability between l-**1**_8_ and l-**2**_8_ was observed upon addition of the methanol-*d*_4_; however, the interactions between the capsules and the solvent differ slightly from the other solvents. Unlike the purely acceptor-type solvent (i.e., acetone-*d*_6_), methanol-*d*_4_ solvates form hydrogen bonds through the C=O acceptors as well as the NH/OH centers. As a result, its disruptive effect cannot be mitigated by unoccupied H-bonding centers, leading to a rapid disassembly of both of capsules at 4 and 6% of the methanol-*d*_4_ for l-**1**_8_ and l-**2**_8_, respectively (Figure S10 in Supporting Information).

The increased stability of the l-**2**_8_ relative to l-**1**_8_ could lead to a better capability for the encapsulation of guest molecules within the internal void of the capsule. The l-**1**_8_ capsule was previously found to be an effective host for C_60_ and C_70_ fullerenes, where the encapsulation of the latter was highly favored.^44^ Guest binding by the octameric capsule l-**2**_8_ in solution was studied by ^13^C NMR spectroscopy (Figure 4f, for ^1^H NMR spectra see Supporting Information). One equivalent of fullerene was first dissolved in TCE-*d*_2_, followed by the addition of 8 equiv of l-**2**, yielding a 1:1 host:guest system. In the ^13^C NMR spectra, free C_70_ shows five NMR resonances at 150.32, 147.76, 146.50, 145.00, and 130.53 ppm in TCE-*d*_2_, while free C_60_ exhibits a single resonance at 142.85 p.p.m (Figure 4f, blue and orange spectra respectively). After the addition of l-**2**_8_ to the C_70_ solution, all ^13^C NMR resonances of C_70_ remained unchanged, indicating no host–guest interaction between l-**2**_8_ and C_70_. However, upon addition of l-**2**_8_ to a C_60_ solution, a new upfield-shifted signal appeared at 140.76 ppm, corresponding to the l-**2**_8_ ⊂ C_60_ complex. Given that the experiment was conducted under homogeneous conditions, the binding constant between C_60_ and l-**2**_8_ could be calculated directly and was found to be 85 m ^–1^—a value comparable to the binding constant of the l-**1**_8_ ⊂ C_60_ complex of 76 m ^–1^ obtained in a similar experiment.^44^ DOSY NMR revealed, the lack of any significant changes in the assembly size upon C_60_ binding, with an observed diffusion coefficient of 8.43 × 10^–11^ m^2^ s^–1^ (Figure S18 in Supporting Information).

We believe that the lack of interaction between l-**2**_8_ and C_70_ is directly related to the slightly smaller size of l-**2**_8_ capsule compared to l-**1**_8_ (11% in DOSY volumes, Figure 1b), as the encapsulation of C_60_ appears to be unaffected. Considering that C_70_ is 15% larger in van der Waals volume compared to its smaller counterpart (646 vs 550 Å^3^),^57^ it seems that the size of a C_70_ fullerene exceeds the limit of l-**2**_8_ breathing motions, preventing its accommodation within the capsule cavity.

### Assemblies from Heterochiral Mixtures

Based on our previous study of l-**1**_8_ and d-**1**_8_ capsules,^44^ we expected the self-assembly of mixtures of the opposite enantiomers of **2** to proceed via narcissistic self-sorting, ultimately yielding enantiopure l-**2**_8_ and d-**2**_8_ capsules from the mixtures of l-**2** and d-**2**. Unexpectedly, the ^1^H NMR spectrum of the l-**2** and d-**2** mixture in 1:1 molar ratio in TCE-*d*_2_ showed a set of new signals, split into four groups (A–D) in 1:1:1:1 integral ratio (Figure 5d). DOSY NMR analysis (Figure S16 in Supporting Information) of this mixture confirmed that all observed signals originated from a single type of assembly, with diffusion coefficient of 1.11 × 10^–10^ m^2^/s (solvodynamic radius of 13.6 Å) which was found to be substantially lower than that observed for l-**2**_8_ capsule. Considering that the *C*_3_-symmetric monomers of l-**2** and d-**2** possess three indivisible homochiral arms and the NMR resonances of their mixture split into four equal groups, the only common multipliers of those numbers are 12, 24, 36 etc., corresponding to tetrameric, octameric, dodecameric (etc.) assemblies, respectively.^58^

**Figure 5:**
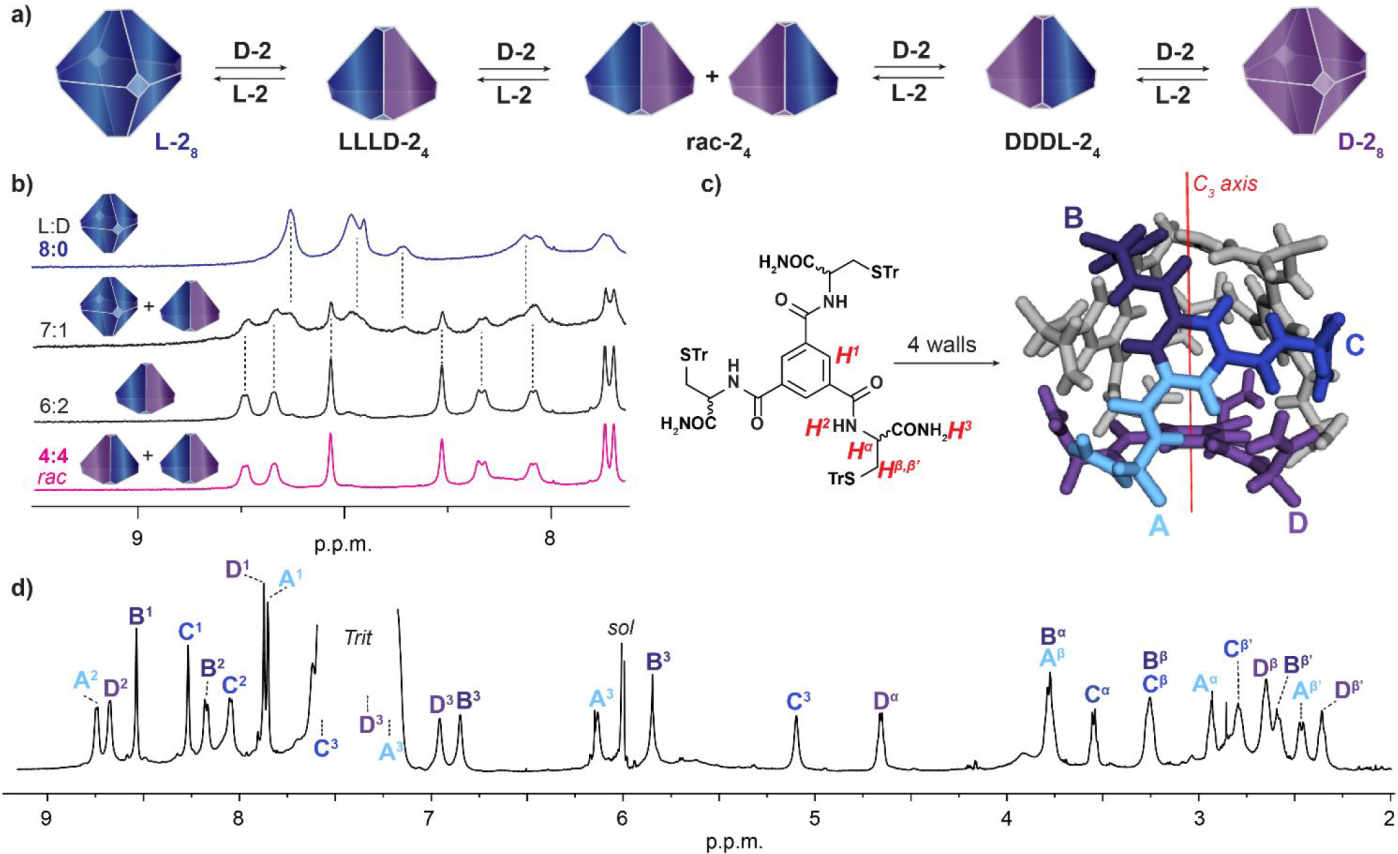
Self-assembly of heterochiral tetrameric assemblies monitored by ^1^H NMR analysis. (a) Schematic representation of the transition between enantiopure octameric assemblies (l-**2**_8_/d-**2**_8_) to tetrameric llld-**2**_4_/ dddl-**2**_4_ assemblies and their racemic mixture. (b) ^1^H NMR spectra (600 MHz TCE-*d*_2_) recorded for l-**2**/d-**2** mixtures at different molar ratios (*C*_total_ = 1.0 × 10^–2^ m, insets show the assembly state observed in solution). (c) Chemical structure of **2** with ^1^H NMR assignment and computational model of llld-**2**_4_ assembly. (d) ^1^H NMR spectrum (700 MHz, TCE-*d*_2_) of llld-**2**_4_ with assigned resonances.

The octameric (and higher) assemblies could be confidently excluded based on the smaller assembly size and the signal pattern in the spectrum. The calculated solvodynamic volume also fell between those observed for l-**3**_2_ and l-**2**_8_ (Figure 1b), aligning well with the expected size of a tetrameric product. To verify this and determine the l-**2**:d-**2** stoichiometry in the assembly, we recorded the ^1^H NMR spectra of such component mixtures in TCE-*d*_2_ at different molar ratios, keeping the total concentration of **2** constant at 1.0 × 10^–2^ m. As shown in Figure 5b, after mixing l-**2** with d-**2** in 7:1 ratio, the spectrum indicated a mixture of the homochiral octamer and a heterochiral assembly. However, when the ratio reached 6:2, the octamer resonances disappeared, leaving only the heterochiral product in solution. The NMR spectrum remains stable up to 2:6 ratio, beyond which the mixture of two assemblies reappeared (Figure S12 in Supporting Information).

This result demonstrates that the self-assembly of the l-**2** and d-**2** mixture proceeds via a social self-sorting mechanism, resulting in quantitative diastereoisomeric selection and yielding a tetrameric assembly with an unexpected 3:1 stoichiometry and low symmetry. The 1:1 mixture of l-**2** and d-**2** forms a racemate of llld-**2**_4_ and dddl-**2**_4_ tetramer isomers (Figure 5b magenta), as evidenced by a similar experiment using Circular Dichroism (Figure S26 in Supporting Information). Notably, a minor offset (≈3%) toward l isomer in the titration data has been observed in both experiments, resulting from a slightly lower enantiopurity of d-**2**, i.e., the component synthesized from the abiotic amino acid synthon. Nevertheless, it has no effect on the observed self-sorting equilibria.

Those findings were further confirmed by a comprehensive 2D NMR analysis. Namely, 2D COSY, ROESY, and ^1^H–^13^C HSQC NMR analyses of llld-**2**_4_ enabled us to assign and match all the resonances within the A, B, C, and D groups.

Additionally, we distinguished the l-**2** and d-**2** components in the assembly based on the exchange peaks (a step-by-step analysis of 2D NMR data has been provided in the Section 1.4 of the Supporting Information). The ^1^H NMR spectrum of llld-**2**_4_ is consistent with the tetrameric assembly that has the lowest possible symmetry for a face-capped tetrahedron: a *C*_3_-symmetric molecule (d-**2**) surrounded by three equal, yet unsymmetrical, molecules of the opposite chirality (l-**2**), resulting in a reduction of the overall symmetry of the assembly from *T* to *C*_3_ (23 to 3 after Hermann–Mauguin).

In line with the experiments for l-**1**_8_ and l-**2**_8_, *rac*-**2**_4_ capsule was titrated with acetone-*d*_6_ to evaluate its stability in solution (Figure S11 in Supporting Information). The assembly was found to be stable up to 10% of acetone (v/v), after which a complex disassembly occurs, most likely to involve solvated homochiral products. Ultimately, the solvated monomer was observed at 50% of acetone-*d*_6_ in TCE-*d*_2_ solution, in line with the l-**2**_8_ capsule.

To understand why the *C*_3_-symmetric llld-**2**_4_ assembly was observed for the specific ratio of enantiomers, while *T* symmetric llll-**2**_4_ and *S*_4_ symmetric lldd-**2**_4_ were never observed, we modeled the tetrameric capsules that can form with different ratios of enantiomers: llll-**2**_4_, llld-**2**_4_, and lldd-**2**_4_ (Figure 6a, insets). The computational models showed that these capsules have 36, 36, and 28 hydrogen bonds per capsule, respectively. Details of the computational models are provided in Section 2 of the Supporting Information. In the llll-**2**_4_ and llld-**2**_4_ capsules, the primary amide acts as both a donor and acceptor, while the secondary amide acts as a donor with the C=O as the acceptor, forming an extended hydrogen-bonded network. However, in the lldd-**2**_4_ capsule, the primary and secondary amides of each monomer cannot align sufficiently to form an extended hydrogen bonding network, which likely explains the absence of the lldd-**2**_4_ experimentally, as it is likely thermodynamically disfavored. The hydrogen bonding networks for both llll-**2**_4_ and llld-**2**_4_ consist of 24 classical acceptor–donor (C=O^···^HN) pairs, along with 12 dynamic pairs between the C=O^···^HN groups and the residual NH groups (Figure 6b). This additional contribution of inter/intramolecular hydrogen bonding via the primary amide in the tetrameric capsule would not exist in the carboxylic acid analogues, which may explain the absence of a carboxylic acid tetrameric capsule.

**Figure 6:**
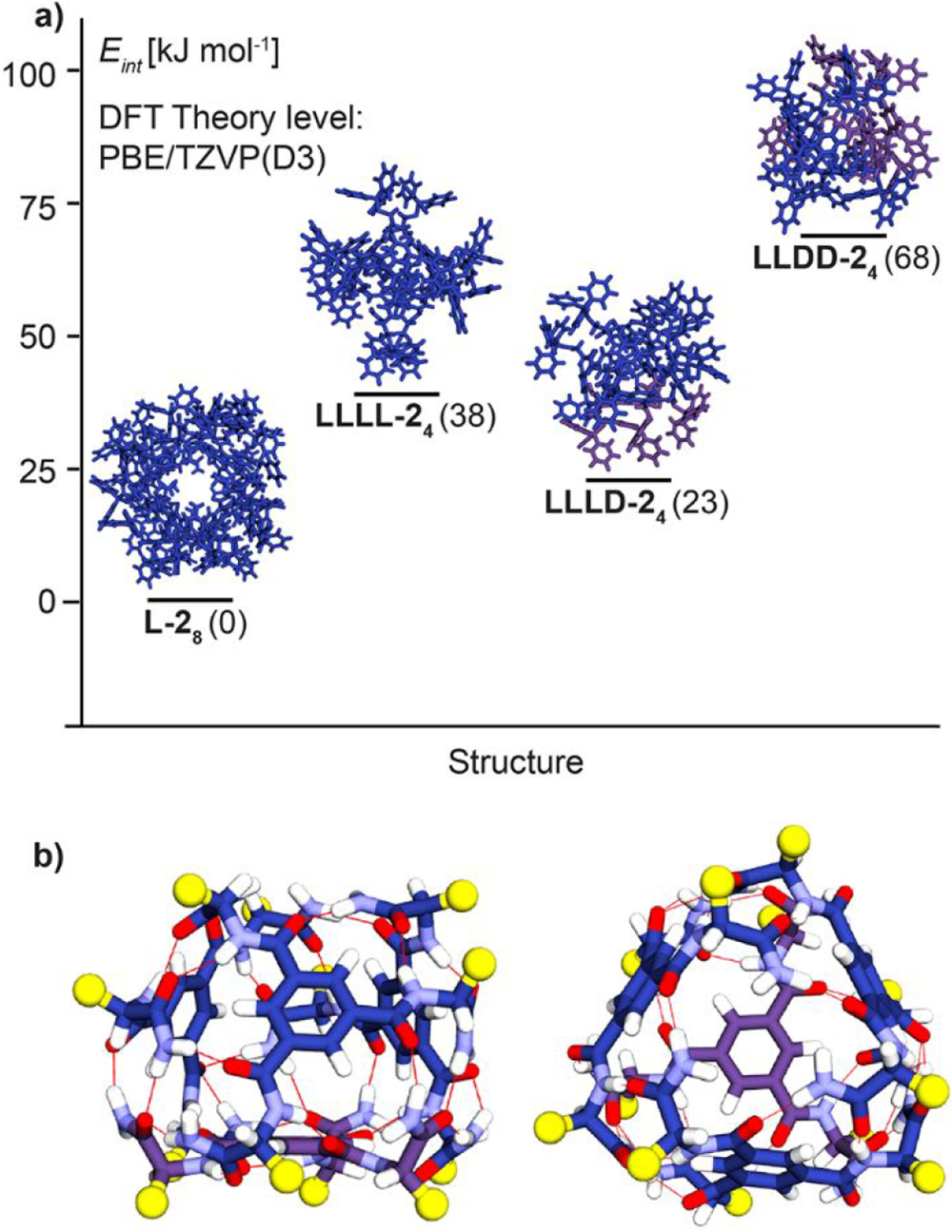
(a) Lowest energy simulated conformation found for each hydrogen-bonded assembly: l-**2**_8_, llll-**2**_4_, llld-**2**_4_, and lldd-**2**_4_, respectively. Monomer molecules of each hydrogen-bonded capsule are colored according to the chirality of the enantiopure building blocks: l-**2** (blue) and d-**2** (purple). (b) DFT model of llld-**2**_4_ with marked hydrogen bonding array (red lines). For clarity, *S*(Tr) units were replaced with methyls (yellow balls).

We calculated the relative DFT energies (at the PBE^59^/TZVP-D3 ^60,61^ level of theory) for the llll-**2**_4,_ llld-**2**_4,_ and lldd-**2**_4_ capsules, relative to the octameric capsule l-**2**_8_ (see Section 2 and Figure S36 in Supporting Information for details). First the structures were optimized with methyl groups in the place of the *S*(Tr)-Cys arms to reduce computational cost. Then the *S*(Tr) units were reincorporated into the structure and optimized further to get the relative energies of the four different capsules (Figure 6a).

The result of the second optimization shows that the lldd-**2**_4_ tetrameric capsule is the most energetically unfavorable, with a relative energy of 68 kJ mol^–1^ compared to l-**2**_8._ This is consistent with experimental results, as lldd-**2**_4_ was never observed experimentally.

The other two tetramers, llll-**2**_4,_ llld-**2**_4_, are 38 kJ mol^–1^ and 23 kJ mol^–1^ higher in energy than the octamer and could therefore be deemed energetically accessible. Therefore, the experimental observation of the llld-**2**_4_ isomer may be explained by configurational entropy effects which favor the formation of the lowest-energy tetramer, even though it is slightly enthalpically unfavored.

### Assemblies from Heterocomponent Mixtures

Following the unexpected chiral self-sorting behavior of l/d-**2** components, we investigated the heterocomponent self-sorting of l-**1**, l-**2**, and l-**3** as well as the guest induced transformations. Initially, three independent equimolar mixtures in TCE-*d*_2_ were prepared: l-**1**:l-**3** (Figure 7a); l-**2**:l-**3** (Figure 7b), and l-**1**:l-**2** (Figure 7c). The analysis showed that l-**3** tends to self-sort narcissistically, yielding independent octameric and dimeric assemblies from the mixtures with both l-**1** and l-**2** (Figure 7a-b). This behavior might be explained by the fact that, unlike l-**1** and l-**2**, the l-**3** component lacks outer carbonyl groups (l-**1** =COOH, l-**2** =CONH_2_ vs l-**3** =CH_2_OH). These outer carbonyl groups play a crucial role in octameric assemblies by acting as acceptors of the C=O^···^H–N hydrogen bonds with the inner secondary amide groups. Four of these hydrogen bonding pairs, along with four C=O^···^HO (or C=O^···^HN for l-**2**) pairs, align four molecules of l-**1** (or l-**2**) around the pores and adjacent walls of the octameric capsules (Figure 1a, c). While the OH group in l-**3** can act as both an acceptor and a donor (Figure 3d), the reduced distance and angles between the hydrogen bonding acceptor and donor sites significantly impact the hydrogen bonding array, making l-**3** incompatible with social assembly.

**Figure 7:**
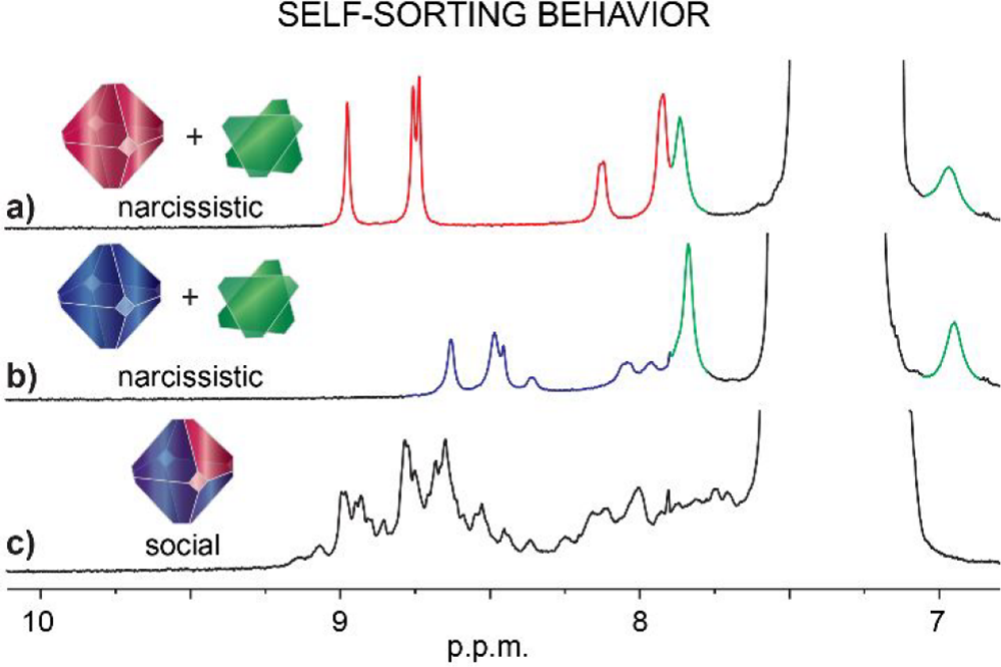
Self-sorting behavior of l-**1**, l-**2**, and l-**3** monitored by ^1^H NMR spectroscopy. ^1^H NMR (600 MHz, TCE-*d*_2_) spectra of 1:1 molar mixtures of: (a) l-**1**+ l-**3**; (b) l-**2**+ l-**3**; (c) l-**1**+ l-**2**.

In contrast, the ^1^H NMR spectrum (Figure 7c) of the equimolar mixture of l-**1** and l-**2** in TCE-*d*_2_ displayed a set of broad and overlapping signals, indicating the formation of hybrid assemblies between l-**1** and l-**2**.

DOSY NMR analysis of this mixture (Figure S17 in Supporting Information) revealed a single diffusion coefficient of 8.62 × 10^–11^ m^2^ s^–1^, corresponding to a hydrodynamic radius of 17.3 Å, which is consistent with the size of hydrogen-bonded octamers of l-**1**_8_ and l-**2**_8_. Due to the structural similarities between l-**1** and l-**2** both in the monomeric and assembled states (particularly in terms of the arrangement of the hydrogen bonding centers), these components can coassemble, ultimately yielding a library of hybrid octameric capsules l-**1**_x_:l-**2**_y_ (Figure 1c). This hybrid formation is driven by social self-sorting. Although these hybrids maintain the overall octahedral topology of the l-**1**_8_ and l-**2**_8_ capsules_,_ the NMR spectrum (Figure 7c) shows no elements of symmetry, as expected for the assembly with a statistical distribution of components on the platonic solid walls.

Given the selective guest recognition of C_60_ and C_70_ fullerenes by octameric capsules (Figure 4f), we also investigated guest-induced transformations between the assemblies. Specifically, llld-**2**_4_ was treated with C_60_ to attempt to steer the heterochiral equilibria toward the formation of homochiral host–guest products (l-**2**_8_ ⊂ C_60_, d-**2**_8_ ⊂ C_60_) via achiral guest. However, the ^13^C NMR spectrum of this mixture revealed that C_60_ remains unbound, and the characteristic splitting pattern in the ^1^H NMR spectrum of llld-**2**_4_ is unaffected by the guest addition (Figure S31 in Supporting Information). This result indicates that the formation of heterochiral products is significantly more favorable than that of homochiral ones, as even guest-induced stabilization cannot overcome the heterochiral equilibrium. Similar to this, l-**1**_x_:l-**2**_y_ octameric capsule does not respond to C_70_ addition, with the entropic effects being the most likely factor of the loss of guest binding ability.

## Conclusions

In conclusion, we have introduced a new class of purely noncovalent capsules based on the cooperative arrays of hydrogen bonds between benzene-1,3,5-tricarboxylic acid derivatives. Incorporating chirality and additional hydrogen bonding sites not only enhanced the thermodynamic stability of these capsules, but more importantly opened the pathway toward diastereoselective formation of heterochiral assemblies with unique 3:1 stoichiometry and low symmetry. The increased stability of the amide-based capsules directly translated into their ability to host guest molecules, with C_60_ being the only guest shown to bind. In contrast, reducing the number of hydrogen bonding pairs simplified the self-assembly product from octamers to a fully symmetric dimer. Furthermore, depending on the chemical composition of the l **-1**, l **-2**, and l **-3** mixtures, the assembly process followed either narcissistic or social pathways, each leading to distinct outcomes. These experimental results, obtained through various spectroscopic techniques, were further supported by exploring the topological landscapes of dimeric, tetrameric and octameric capsules through atomistic modeling and DFT calculations, which helped explain the selectivity of either narcissistic or social pathways at an energetic level. We believe that these newly designed capsules will pave the way for developing nonsymmetrical hydrogen-bonded systems—a realm previously dominated by metallosupramolecular and covalent systems. Notably, the components presented in this study are based on the well-established supramolecular synthon, i.e., benzene-1,3,5-tricarboxylic acid, which offers almost unlimited potential for simple chemical modifications. This versatility allows the systems to be easily adapted for a wide range of applications.

## Supplementary Material



## Notes

Synthesis, characterization data, supplementary spectra, and computational details (PDF)

## #

Department of Chemistry, University College London, London, WC1H 0AJ, U.K
