# Supplementary material for: Chirality-Assisted Self-Assembly of Low-Symmetry Noncovalent Capsules with Quantitative Diastereoisomeric Selection
Source: J Am Chem Soc. 2025 Aug 15;147(34):31270–9. doi: 10.1021/jacs.5c10523 (PMC12395406; doi:10.1021/jacs.5c10523)
Supplement: Supplementary file 1 [file ja5c10523_si_001.pdf]

Supporting information

for

Chirality-Assisted Self-Assembly of Low Symmetry Non-Covalent Capsules with Quantitative Diastereoisomeric Selection

Grzegorz Markiewicz,* [a,b] Xujun Qiu,[a,b] Gokay Avci,[c] Emma H. Wolpert,[c] Kim E. Jelfs,[c] Jeremy K. M. Sanders,[d] and Artur R. Stefankiewicz*[a,b]

[a] Center for Advanced Technologies, Adam Mickiewicz University, Uniwersytetu Poznańskiego 10, 61-614 Poznań, Poland.
[b] Faculty of Chemistry, Adam Mickiewicz University, Uniwersytetu Poznańskiego 8, 61-614 Poznań, Poland.
[c] Department of Chemistry, Imperial College London Molecular Science Research Hub, White City Campus, Wood Lane, London W12 0BZ, United Kingdom.
[d] Yusuf Hamied Department of Chemistry, University of Cambridge, Lensfield Road, Cambridge CB2 1EW, United Kingdom.

Table of Contents

Table of Contents 1
1. Experimental Procedures 2
1.1 General 2
1.2 Synthesis and characterization of 2 and 3 4
1.3 FT-IR spectra 9
1.4 NMR Titrations 11
1.5 2D NMR data and analysis 13
1.6 Fullerene encapsulation 23
2. Computational Procedures 25
3. References 30

1. Experimental Procedures

1.1 General

All the commercially available reagents and solvents were used without any further purification unless otherwise stated. Thin layer chromatography was performed using TLC plates from Sigma-Aldrich® with fluorescence indicator 254 nm and monitored with a UV-light irradiation (254 nm and 365 nm). Column chromatography were conducted using silica gel with average pore size 60 Å (52-73 Å) from Sigma-Aldrich®.

NMR spectra were recorded on Bruker Avance III HD 400 MHz (5 mm BBFO probe), Bruker Avance III HD 600 MHz (5 mm BBFO probe) and Bruker Avance III 700 MHz (5 mm BBI probe) spectrometers at 298 K. Chemical shifts(δ) in ^{1}H NMR and ^{13}C NMR spectra were referenced on solvent residual peaks as follows: DMSO-d_6 (δH = 2.50 ppm, δC = 39.52 ppm), TCE-d_2 (δH = 6.00 ppm, δC = 73.78 ppm).

DOSY NMR spectra were recorded using 2D LEDbp-pulse sequence (ledbpgp2s). Δ and δ were optimised with 1D sequence (ledbpgp2s1d) to achieve sufficient signal attenuation at 95% gradient strength. Probe-specific Gradient constant was calibrated using Bruker standard sample (doped water, Bruker P/N Z10906), and cross-checked against DMSO-d_6 sample, in accordance with Bruker *almanac*. All DOSY experiments were recorded at T = 298 K. Sample temperatures were equilibrated for at least 20 min prior to the measurement to avoid temperature gradient within NMR tubes. Solvent residual peaks were used as the internal standards of diffusion rates. TCE-d_2 viscosity was determined via DOSY NMR using TMS as an internal standard. Diffusion coefficients were obtained from *T1/T2* analysis (Bruker TopSpin 4.0) and cross-checked with Bayesian transformation (MNova 11.0).

Solvodynamic radii were calculated using Einstein-Stokes equation for spherical objects, as follows:

$$D = \frac{k_b T}{6\pi\eta r}$$

The following solvent viscosities were used for calculations:

$$\eta DMSO = 1.99 \text{ mPa.s}$$

$$\eta \text{TCE} = 1.46 \text{ mPa.s}$$

2D NMR spectra were recorded with the following Bruker pulse sequences:

^{1}H-^{1}H COSY (cosygpppqf),^{1}H-^{13}C HSQC (hsqcedetgpsisp2.3); ^{1}H-^{1}H ROESY (roesyphpp.2).

ESI-MS spectra were recorded on Bruker Impact HD Q-TOF spectrometer in positive ion mode.

CD spectra were recorded on Jasco Co. (Japan) J-1500 CD spectropolarimeter, operating at 1 nm bandwidth and 400 nm/min scanning speed. Sample temperature was controlled with a Peltier-type variable-temperature unit (Jasco Co., PTC-510). Measurements were performed in solutions using quartz cuvettes (Hellma GMbH, Germany) with 1 mm optical path. Spectra of the pure solvents were used as the baselines.
Thermodynamic analysis of L-$\mathbf{3}_2$ was performed following the procedure described in our previous work.[1] The dimerization equilibrium constant *K* was defined as follows:

$$K = \frac{[L{-}3_2]}{[L-3]^2}$$

Where [L-$\mathbf{3}_2$] and [L-**3**] denote the concentrations of the individual species at equilibrium, and the total concentration of the sample C_T is defined as:

$$C_T = 2[L{-}3_2] + [L{-}3_2]$$

ΔCD at λ = 220 nm was plotted as a function of temperature *T* and fitted using the *T*-dependent *equal-K* (isodesmic) assembly model.[2] The thus obtained plot was then normalized within aggregation degree range α = 0.0 and α = 1.0. With the assumption, that the CD at α = 0.0 represents the molar ellipticity of a monomer, and the CD at α =1.0 represents the molar ellipticity of a dimer.
The individual concentrations of [L-$\mathbf{3}_2$] and [L-**3**] and subsequently *K* at particular temperature *T*, were calculated from the observed α and material distribution. *K* was determined for various temperatures *T* in 270-360 K range, and subsequently *lnK* was plotted as a function of *1/T* according to the linear Van't Hoff relation. *ΔH* and *ΔS* were determined from the slope and intercept respectively.

FT-IR spectra were recorded on Jasco FT/IR-4700 spectrometer in the airtight CaF_2 cuvette of 0.2 mm pathlength. Spectra of the pure solvents were used for subtraction.

1.2 Synthesis and characterization of 2 and 3

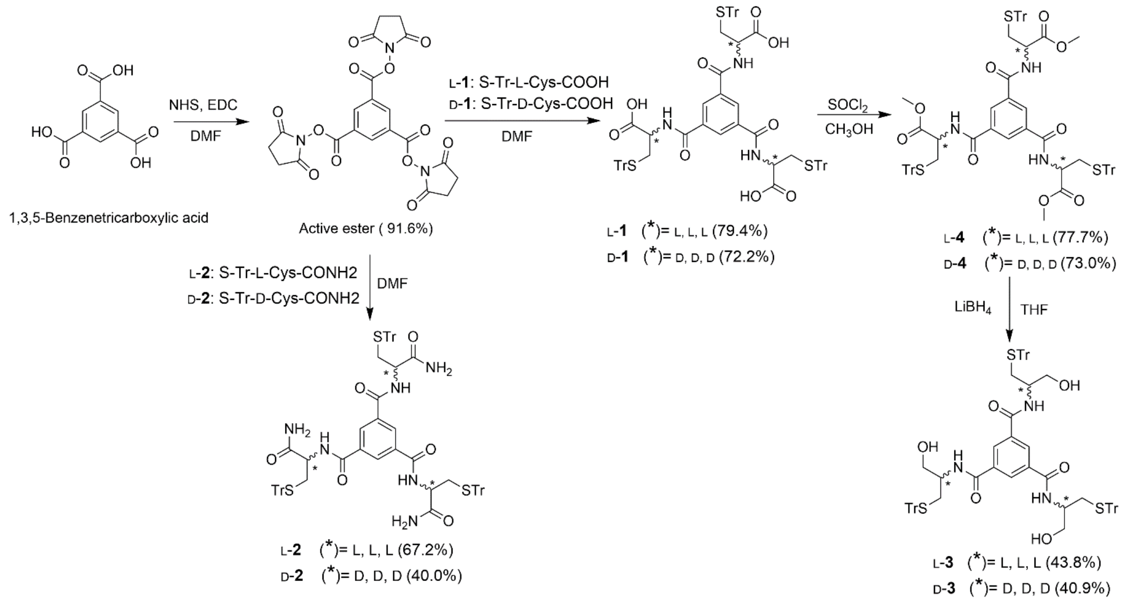

Scheme S1. Synthesis of L-**2**, D-**2**, L-**3** and D-**3** (Tr=trityl).

Active ester, L-**1,** and D-**1** were synthesized according to the procedure described in our previous work[3], *S*-Tr-D-Cys-CONH$_2$ was prepared following the literature procedure starting from *S*-Tr-D-Cys-COOH[4].

L-**2**: To a solution of active ester (0.125 g, 0.25 mmol) in anhydrous DMF (7 mL), *S*-Tr-L-Cys-CONH$_2$ (0.543 g, 1.5 mmol) and triethylamine (0.2 mL, 1.5 mmol) were added. After overnight reaction at the room temperature the solvent was removed to afford brown oil, which was dissolved in acetone (4 mL) and added dropwise into 1 M HCl (40 mL) under vigorous stirring. The white precipitate was filtered off, washed with H_2O (20 mL) and dried under the vacuum. Afterwards, the above product was dissolved in DCM (2 mL) and poured into *n*-hexane (30 mL) while stirring to afford white precipitate. After filtering and drying, the raw product was obtained, which was further purified by column chromatography (CC condition: CH_3OH: DCM= 1:19) to obtain pure L-**2** as white powder (yield 0.21 g, 67%). ^{1}H NMR (600 MHz, DMSO-d_6) δ 8.81 (d, J = 8.3 Hz, 3H), 8.48 (s, 3H), 7.45 (s, 3H), 7.32 - 7.21 (m, 45H), 7.18 (s, 3H), 4.56 (td, J = 8.5, 5.8 Hz, 3H), 2.56 - 2.51 (m, 6H). ^{13}C NMR (151 MHz, DMSO-d_6) δ 171.52, 165.19, 144.30, 134.07, 129.49, 129.09, 128.06, 126.75, 65.96, 52.41, 33.79. ESI-MS: Calc. $[M+H]^+$ = 1243.428, $[M+Na]^+$= 1265.410 *m/z*. Found: $[M+H]^+$ 1243.427, $[M+Na]^+$ 1265.409 *m/z.*

D-**2**: D-**2** was synthesized according to L-**2** procedure using *S*-Tr-D-Cys-CONH$_2$ (yield 0.12 g, 40%). ^{1}H NMR (600 MHz, DMSO-d_6) δ 8.82 (d, J = 8.4 Hz, 3H), 8.49 (s, 3H), 7.45 (s, 3H), 7.34 - 7.21 (m, 45H), 7.18 (s, 3H), 4.57 (td, J = 8.4, 5.8 Hz, 3H), 2.57 - 2.52 (m, 6H). ^{13}C NMR (151 MHz, DMSO-d_6) δ 171.52,

165.19, 144.30, 134.08, 129.50, 129.09, 128.06, 126.75, 65.96, 52.41, 33.79. ESI-MS: Calc. $[M+H]^+$ = 1243.428, $[M+Na]^+$= 1265.410 *m/z*. Found: $[M+H]^+$ 1243.425, $[M+Na]^+$ 1265.407 *m/z.*

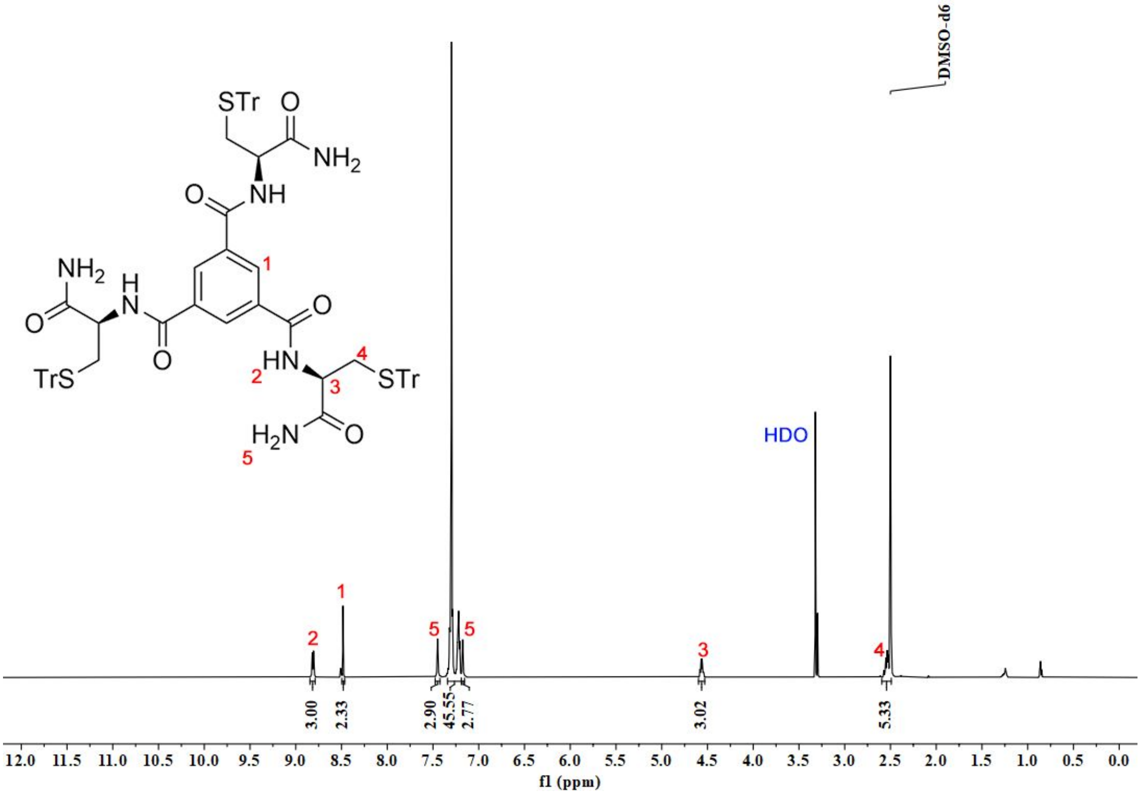

Figure S1. ^{1}H NMR spectrum (DMSO-d_6, 600 MHz) of L-**2**.

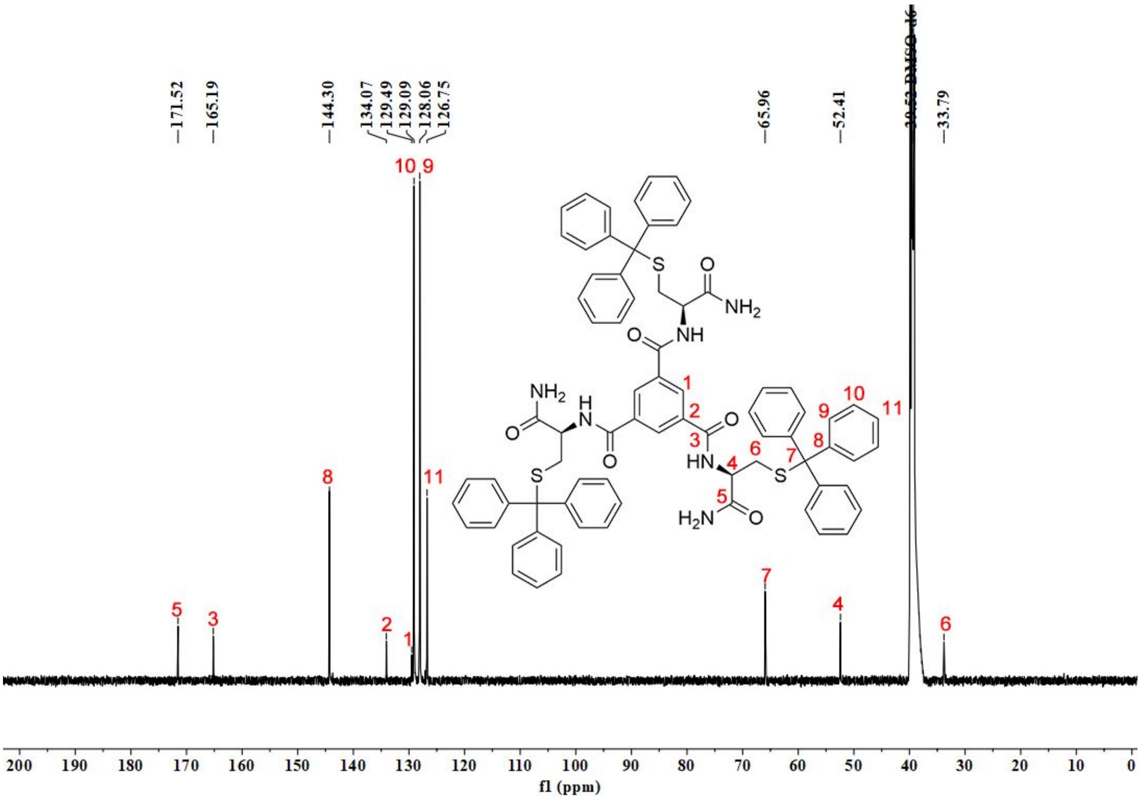

Figure S2. ^{13}C NMR spectrum (DMSO-d_6, 150 MHz) of L-**2**.

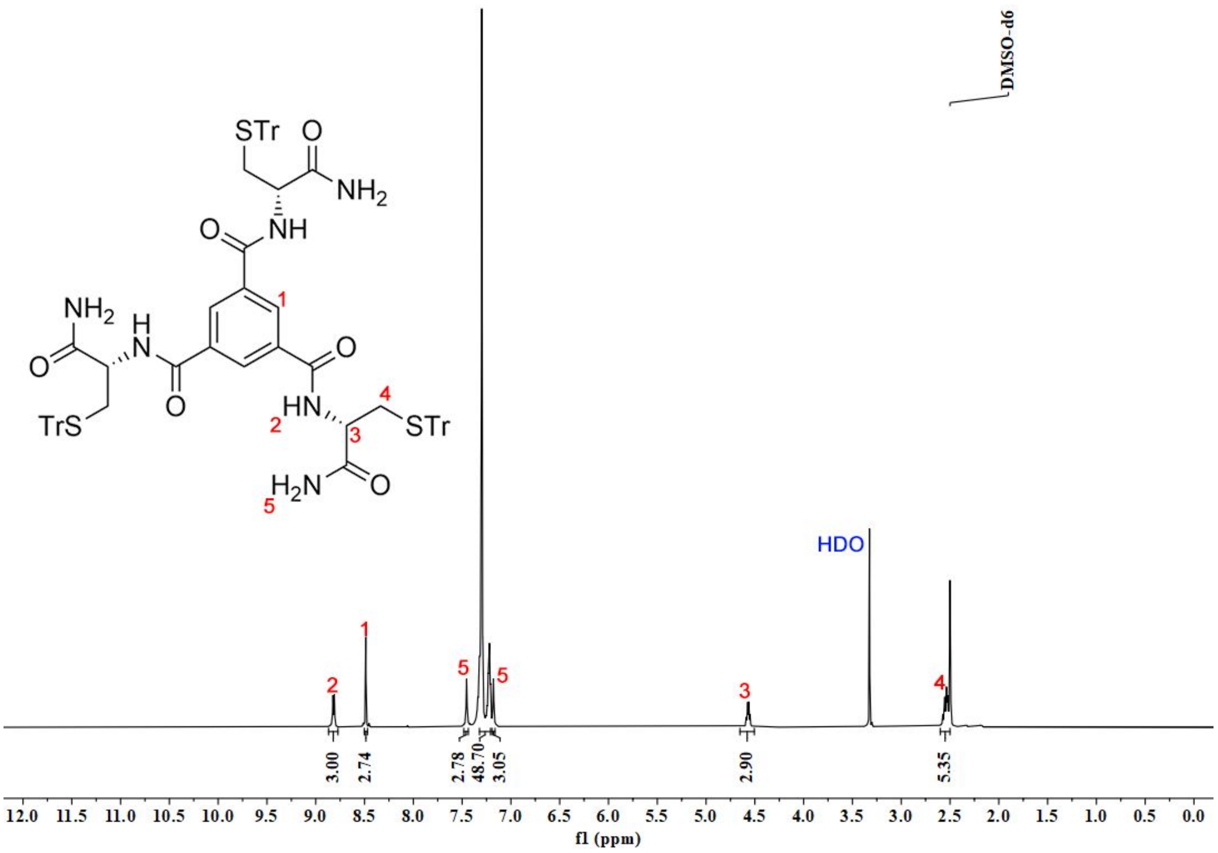

Figure S3. ^{1}H NMR spectrum (DMSO-d_6, 600 MHz) of D-**2**.

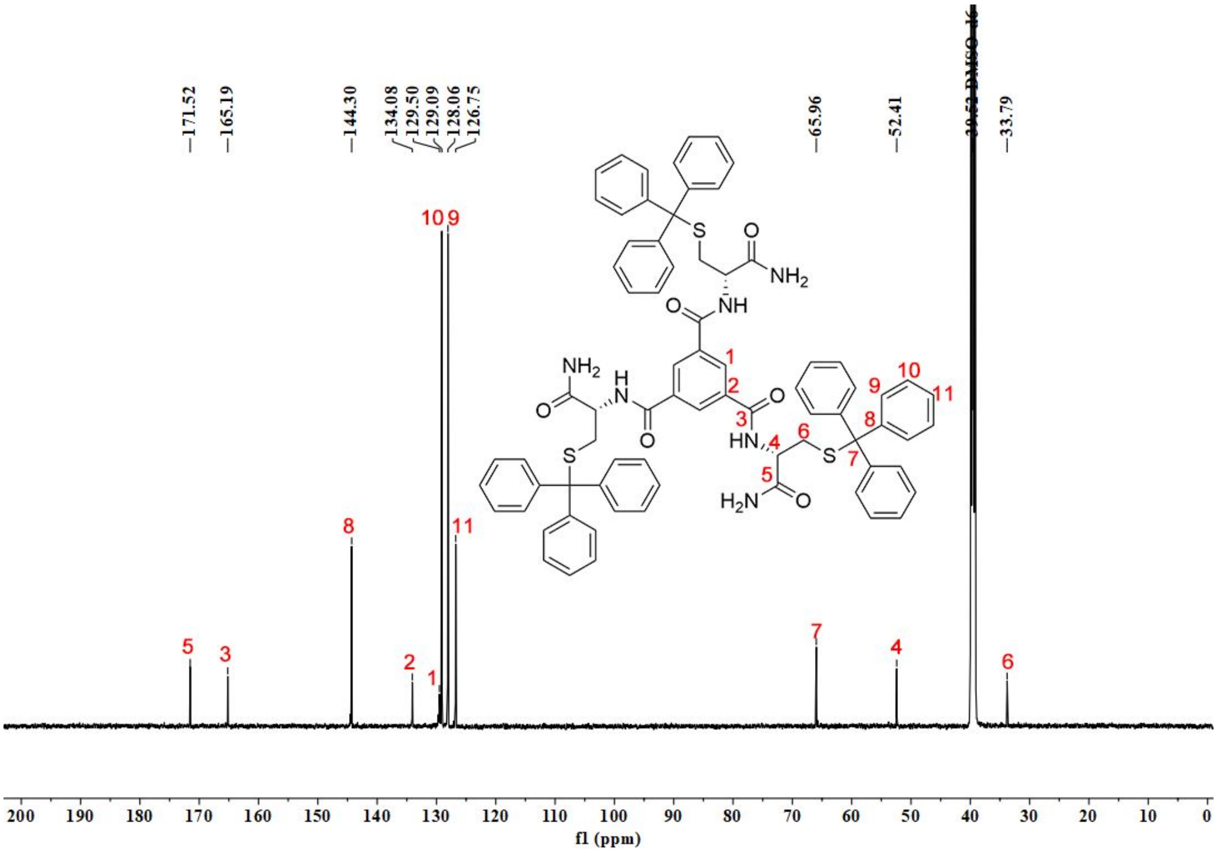

Figure S4. ^{13}C NMR spectrum (DMSO-d_6, 150 MHz) of D-**2**.

L-**3**: **Step 1**: To a solution of L-**1** (250 mg, 0.2 mmol) in methanol (20 mL), thionyl chloride (1 mL, 14 mmol) was added drop wise. The mixture was stirred at room temperature overnight. After removing the volatiles via vacuum, the residue was treated with diethyl ether (20 mL) to afford brown precipitate. After filtering off, the raw methyl ester L-**4** was dried in vacuum, resulting in brown powder (yield 200 mg, 78%), which was used in the next step without further purification. **Step 2:** To a solution of L-**4** (100 mg, 0.08 mmol) in THF (25 mL), $LiBH_4$ (20 mg, 0.9 mmol) was added in five portions while stirring at 0 °C. The reaction was left overnight at room temperature. After removing the solvents, brown oil-wise residue was afforded. The crude product L-**3** was purified by column chromatography (CC condition: CH_3OH: DCM=1: 9) (yield 42 mg, 44%). ^{1}H NMR (600 MHz, DMSO-d_6) δ 8.53 (d, J = 8.4 Hz, 3H), 8.43 (s, 3H), 7.35 - 7.18 (m, 45H), 4.79 (t, J = 5.7 Hz, 3H), 4.07 (td, J = 8.0, 4.4 Hz, 3H), 3.38 (dt, J = 11.0, 5.6 Hz, 3H), 3.31 - 3.28 (m, 3H), 2.46 - 2.37 (m, 6H). ^{13}C NMR (151 MHz, DMSO-d_6) δ 165.18, 144.49, 134.79, 129.11, 128.82, 128.00, 126.68, 65.85, 62.58, 51.35, 33.15. ESI-MS: Calc. $[M+H]^+$ = 1204.442 $[M+Na]^+$= 1226.424 *m/z*. Found: $[M+H]^+$ 1204.442, $[M+Na]^+$ 1226.420 *m/z*.

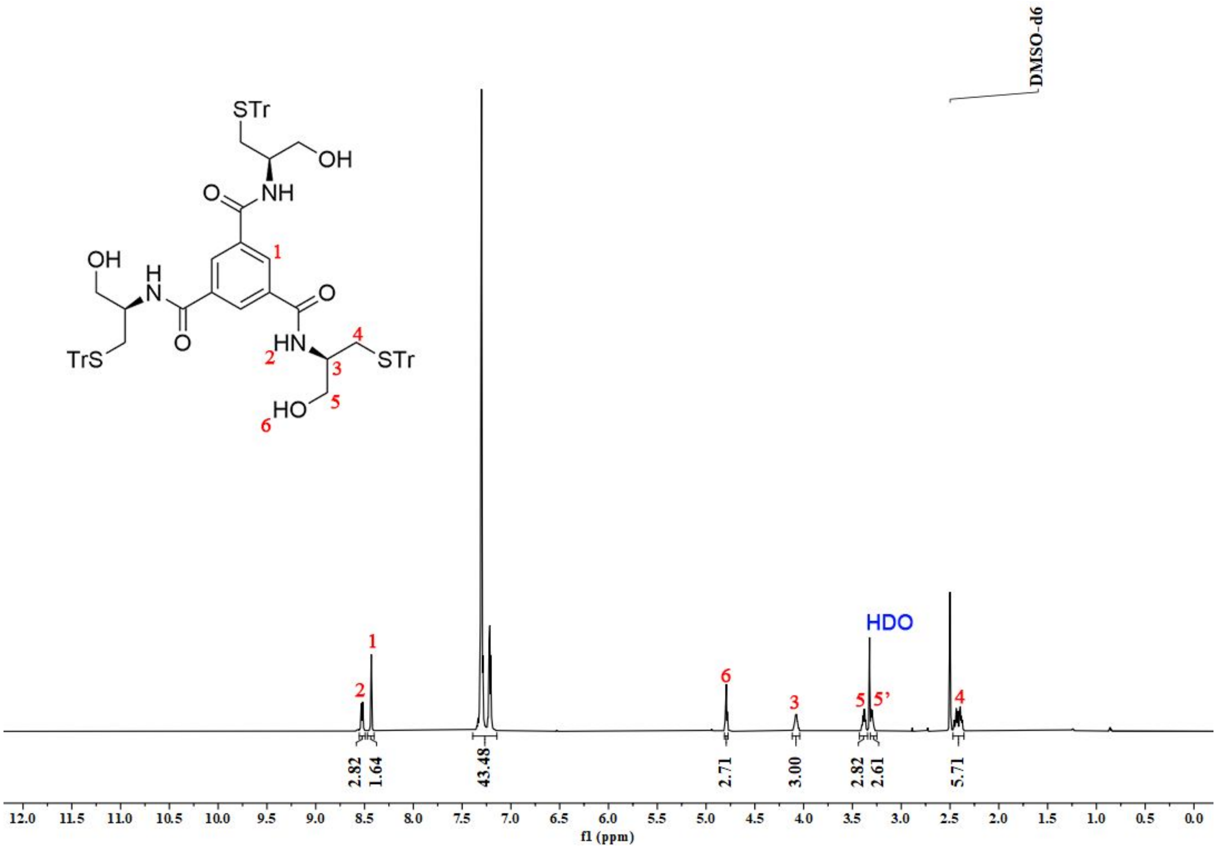

Figure S5. ^{1}H NMR spectrum (DMSO-d_6, 600 MHz) of L-**3**.

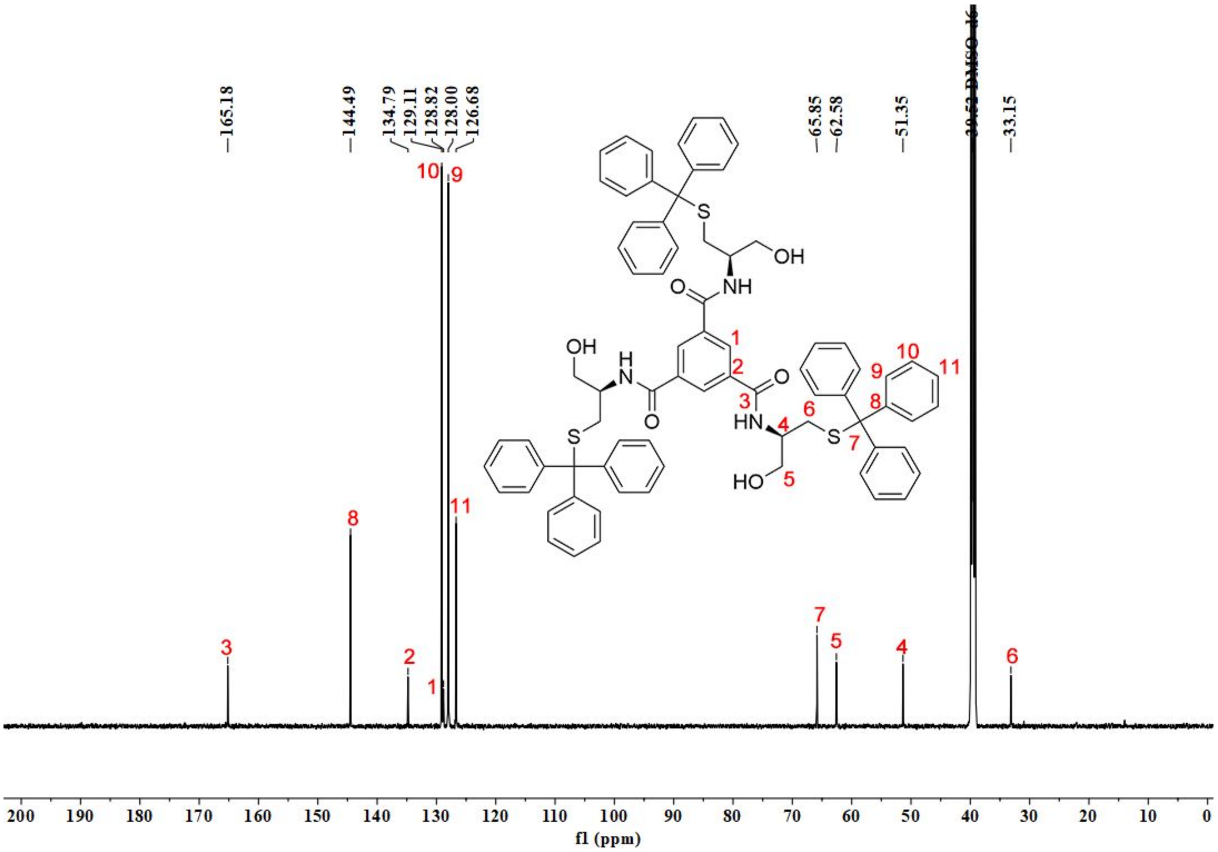

Figure S6. ^{13}C NMR spectrum (DMSO-d_6, 150 MHz) of L-**3**.

1.3 FT-IR spectra

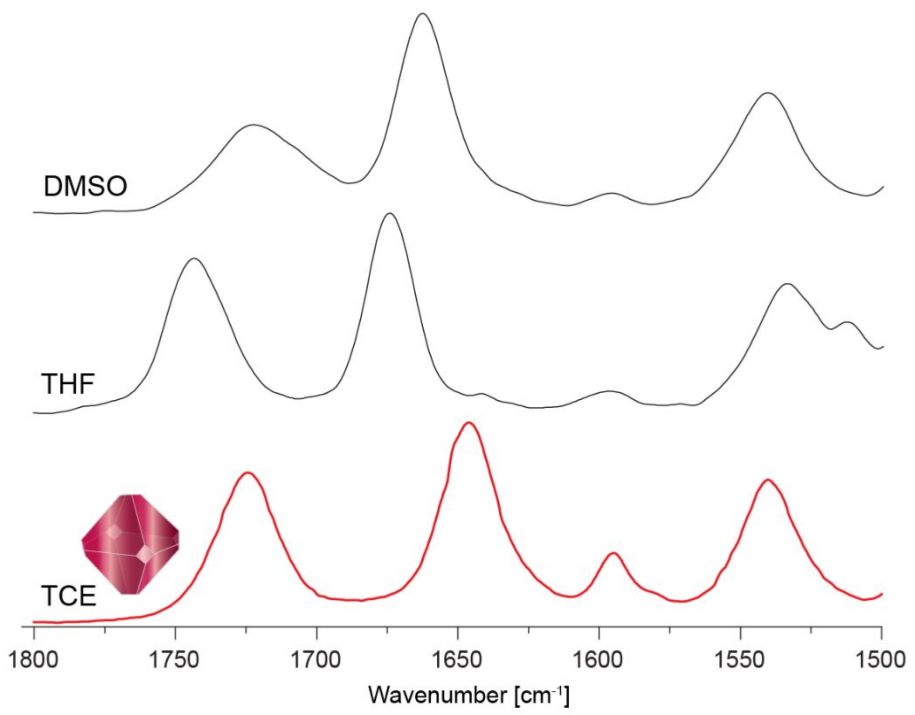

Figure S7. FT-IR spectra of L-**1** (*C* = 1.0×10^{-2} M) recorded in DMSO (top), THF (middle), TCE (bottom). Spectra of the pure solvents were used for subtraction.

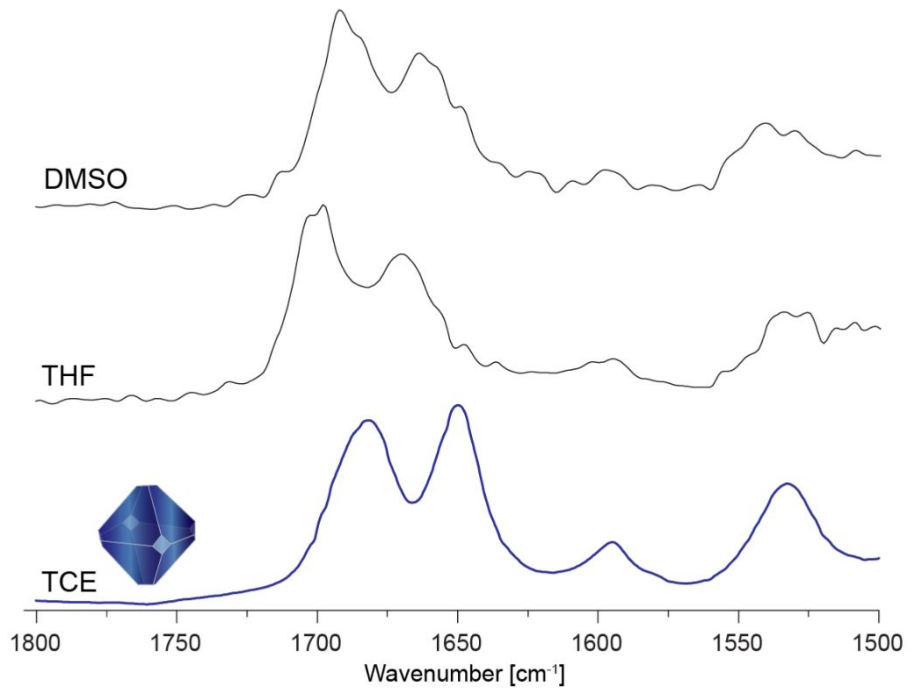

Figure S8. FT-IR spectra of L-**2** (*C* = 1.0×10^{-2} M) recorded in DMSO (top), THF (middle), TCE (bottom). Spectra of the pure solvents were used for subtraction.

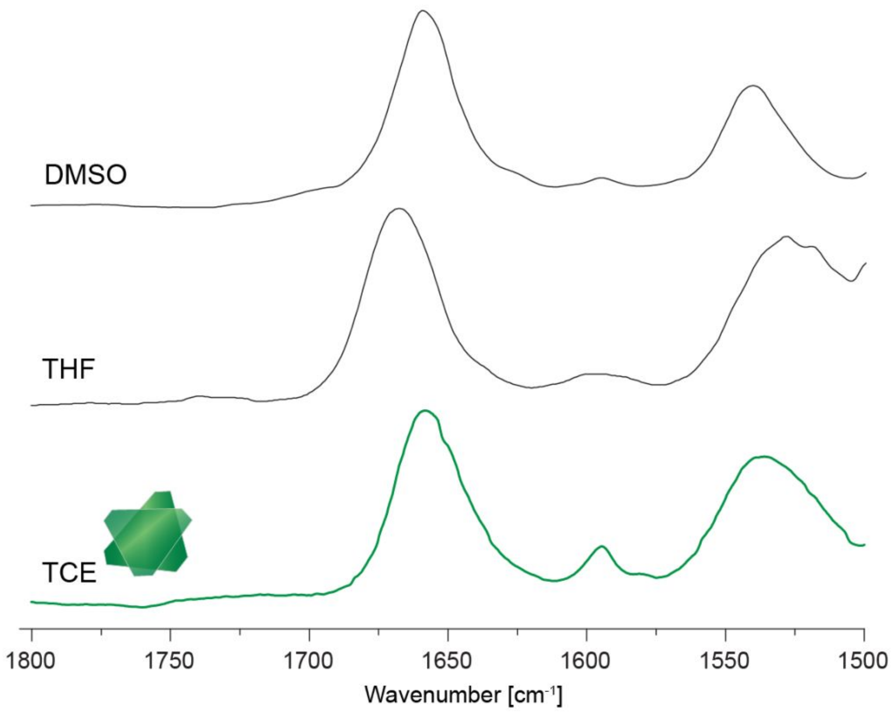

Figure S9. FT-IR spectra of L-**2** (C = 1.0×10^{-2} M) recorded in DMSO (top), THF (middle), TCE (bottom). Spectra of the pure solvents were used for subtraction.

1.4 NMR Titrations

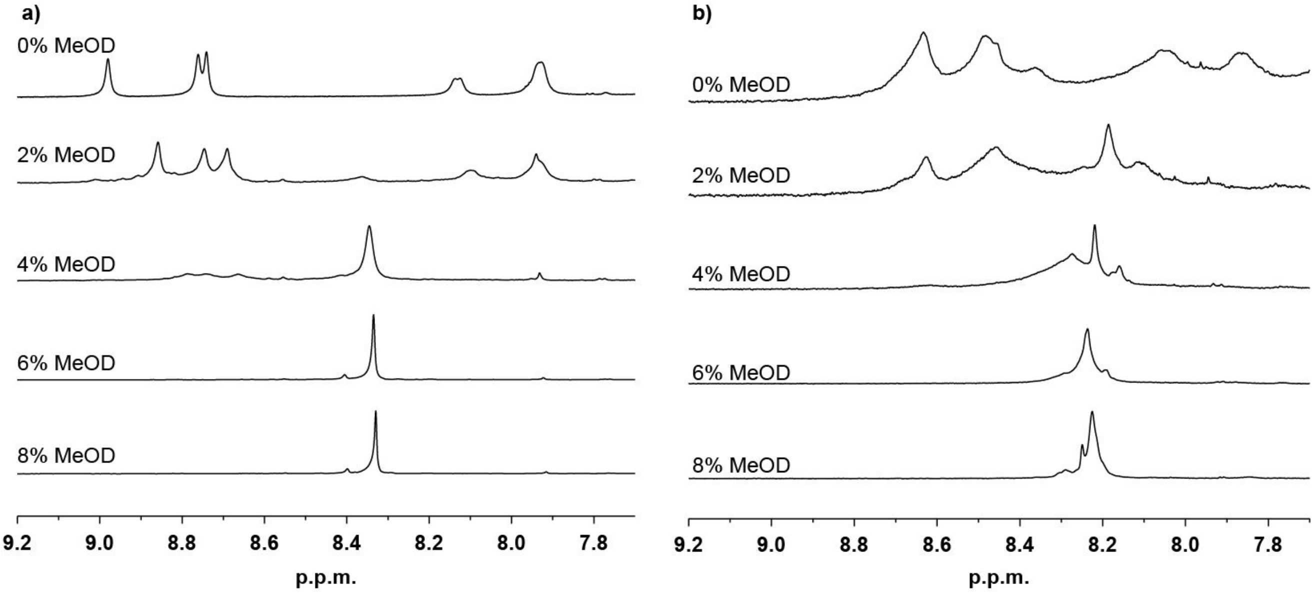

Figure S10. ^{1}H (600 MHz TCE-d_2) disassembly spectra of: a) L-**1**$_8$ upon addition of methanol-d_4. b) L-**2**$_8$ upon addition of methanol-d_4.

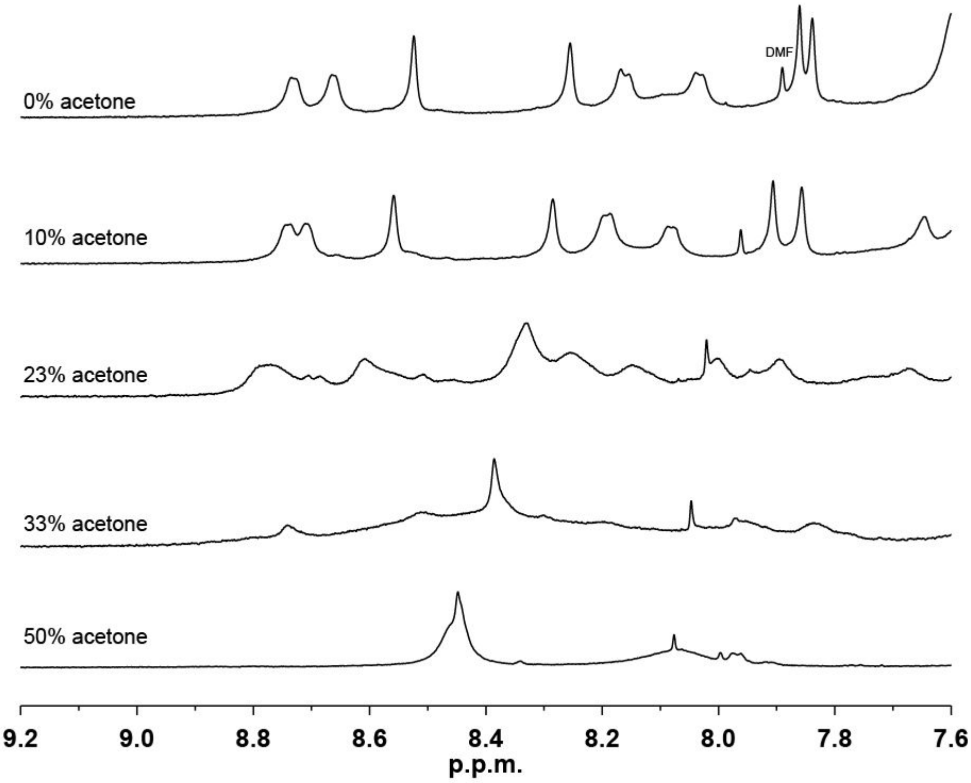

Figure S11. ^{1}H (600 MHz TCE-d_2) disassembly spectra of: *rac*-**2**$_4$ upon addition of acetone-d_6.

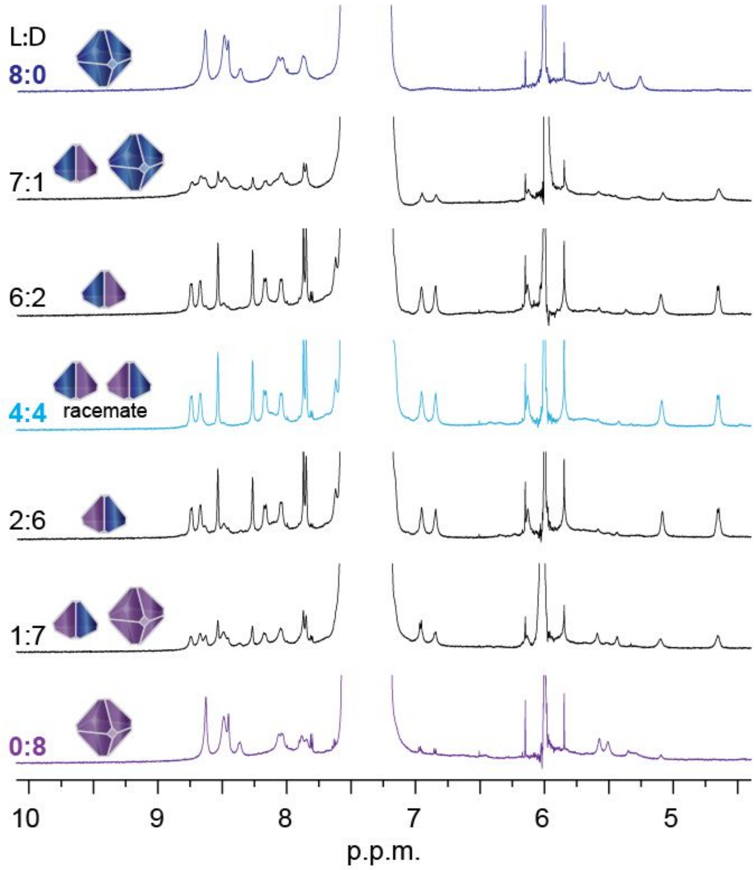

Figure S12. ^{1}H NMR spectra (600 MHz TCE-d_2) recorded for L-**2**/D-**2** mixtures at different molar ratios (C_{total} = 1.0×10^{-2} M, inserts show the assembly state observed in solution)

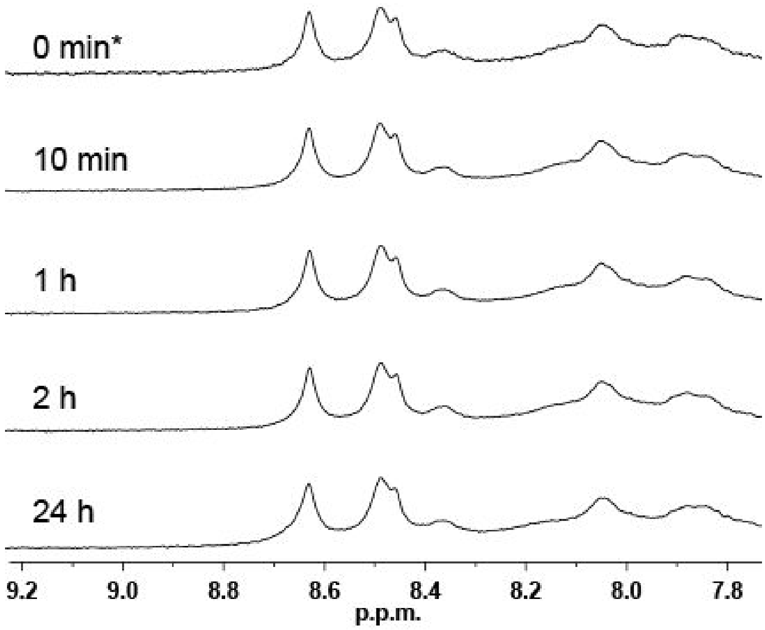

Figure S13. Exemplary time-dependent ^{1}H NMR spectra (400 MHz TCE-d_2) recorded for L-$\mathbf{2}_8$. (C_{total} = 1.0×10^{-2} M). * 0 min denotes the 16-scan spectrum recorded immediately after dissolution (≈30 sec) of the solid material in TCE-d_2 at 298 K.

1.5 2D NMR data and analysis

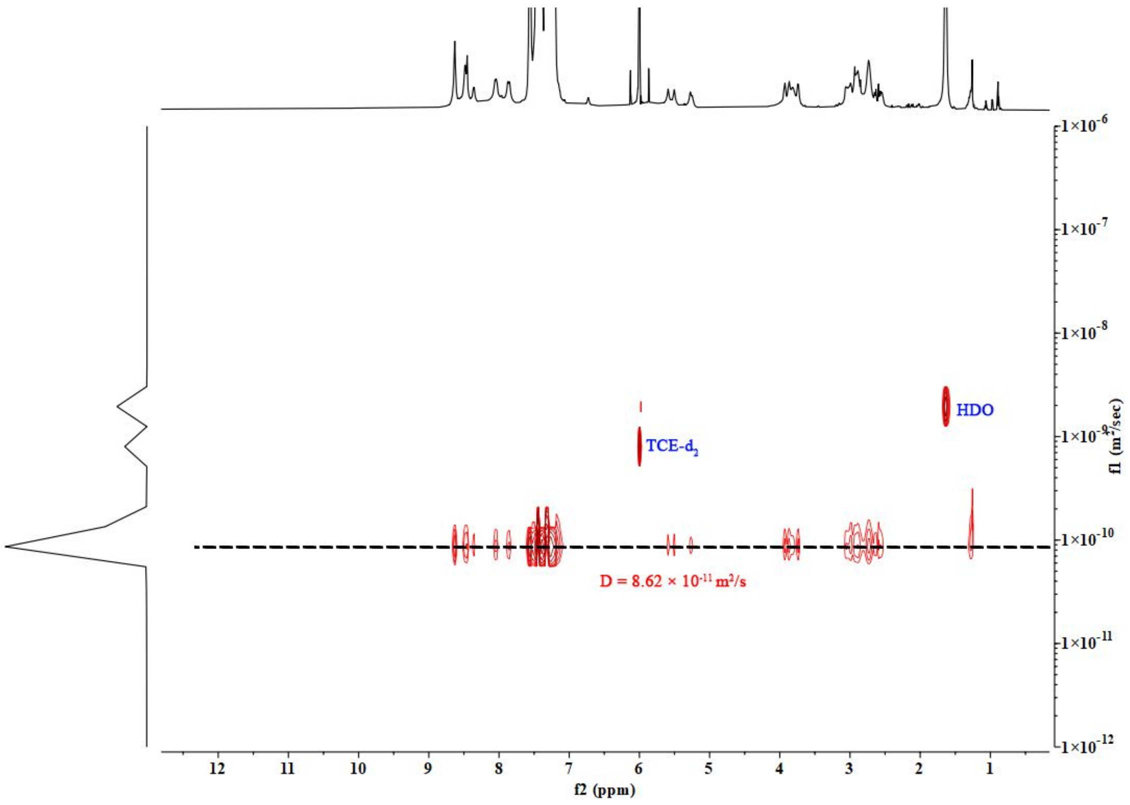

Figure S14. ^{1}H (700 MHz TCE-d_2) DOSY spectrum of L-$\mathbf{2_8}$.

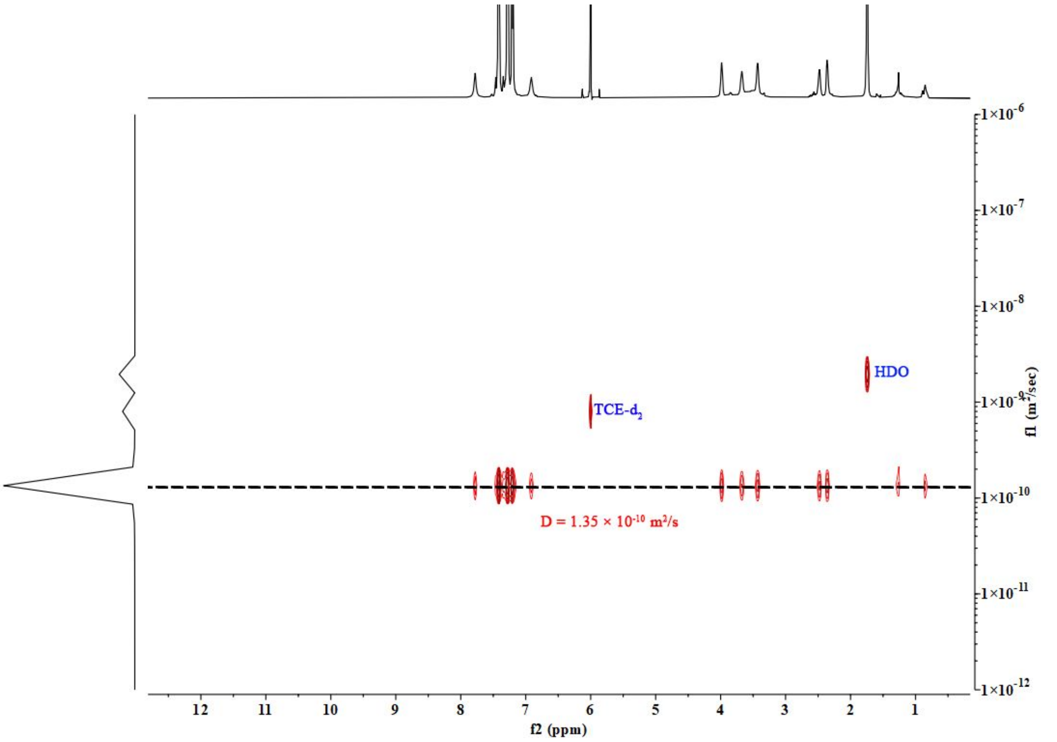

Figure S15. ^{1}H (700 MHz TCE-d_2) DOSY spectrum of L-$\mathbf{3_2}$.

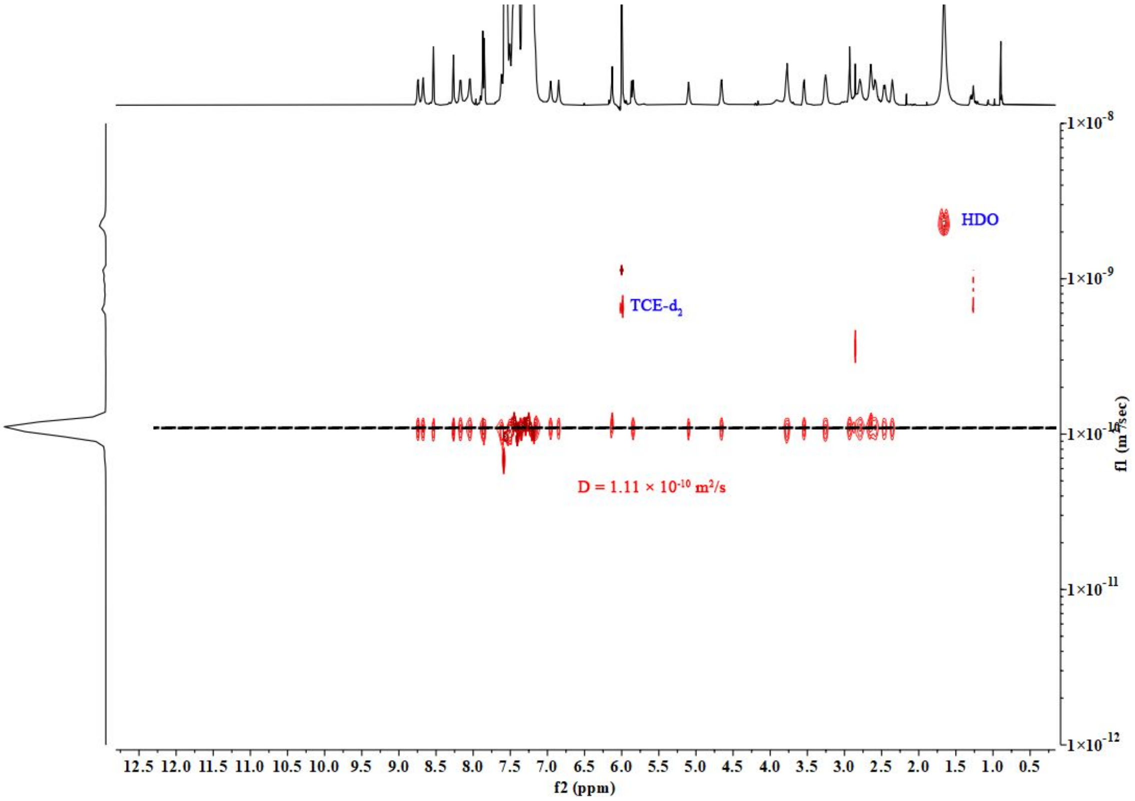

Figure S16. ^{1}H (700 MHz TCE-d_2) DOSY spectrum of *rac*-$\mathbf{2}_4$.

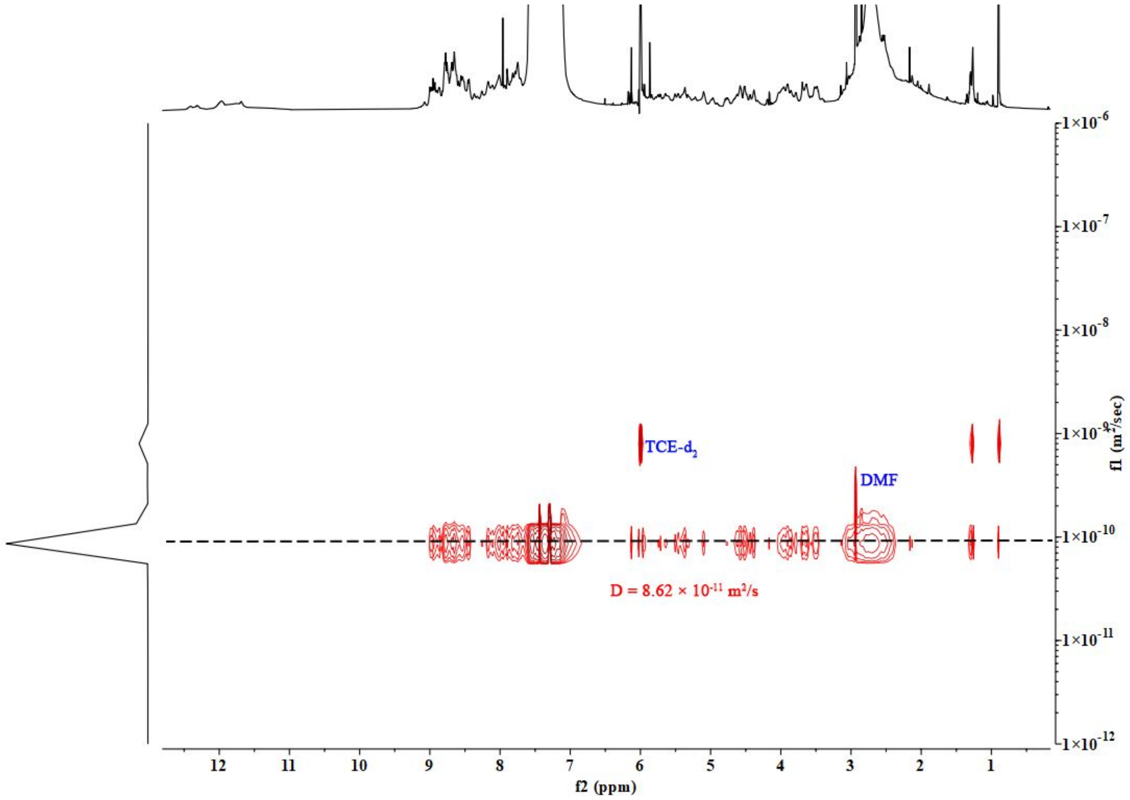

Figure S17. ^{1}H (700 MHz TCE-d_2) DOSY spectrum of L-**1**+L-**2** in 1:1 ratio.

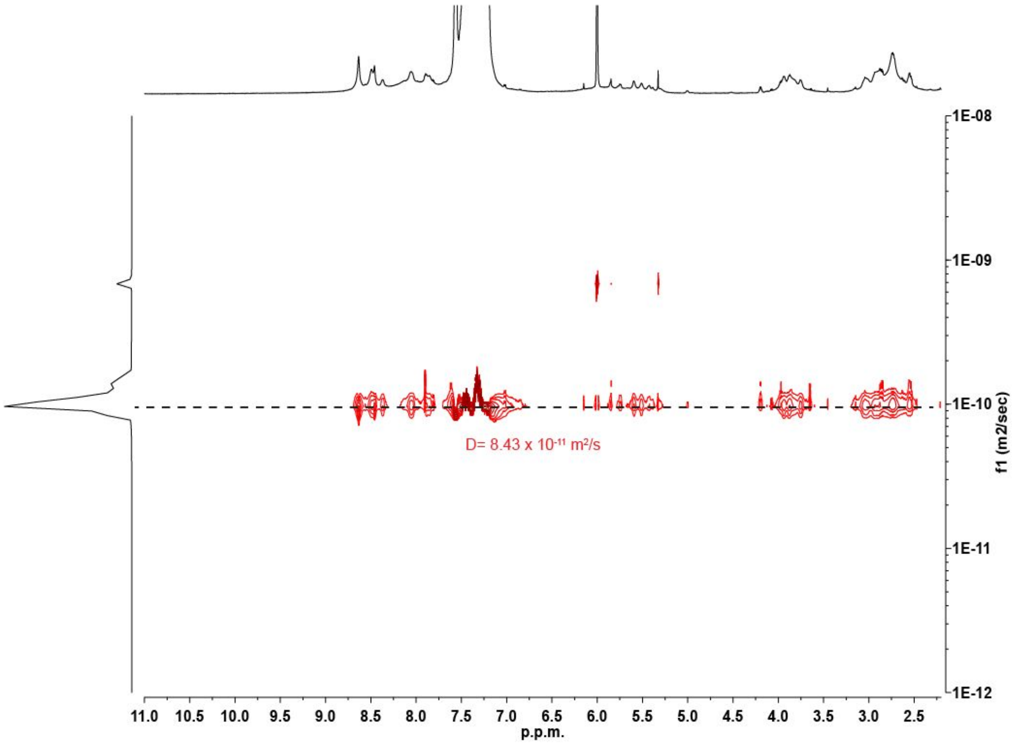

Figure S18. ^{1}H (600 MHz TCE-d_2) DOSY spectrum of L-**2**$_\mathbf{8}$ <C_{60} complex.

LLLD-2$_4$

Despite the relative simplicity of the chemical structure of **2**, the ^{1}H NMR spectrum of the mixture of L/D-**2** recorded in TCE-d_2 shows very complex symmetry. To assign the peaks, a detailed analysis of 2D NMR spectra was performed, as follows:

1. First the aromatic region (9.0-7.5 ppm) was analysed. Here, two signals were expected: aromatic **C-H (peak 1)** and amido **N-H (peak 2)**.

(Note: *Albeit CH and NH peaks could be distinguished based on signal width and multiplicity, we don't consider this a reliable tool in this case, because the entire spectrum shows strong broadening effects due to self-assembly.)*

Peaks 1 and **peaks 2** can be distinguished based on ^{1}H-^{13}C HSQC and ^{1}H-^{1}H COSY experiments. Namely, **peaks 1** were expected to show $^1J_{C-H}$ corelations with aromatic resonances (120-140 ppm) while **peaks 2** were expected to show $^3J_{H-H}$ corelations with **α-peaks** in the aliphatic region (2-5 ppm).

Indeed, the positions of four **peaks 1** (at 8.54, 8.27, 7.88, 7.85 ppm) and four **peaks 2** (at 8.75, 8.67, 8.17, 8.05 ppm) were obtained from ^{1}H-^{13}C HSQC and ^{1}H-^{1}H COSY experiments, respectively.

For clarity, those four groups were named A, B, C, D.

	H^1	H^2	$H^α$	$H^β$	H^3
**A**					
**B**	8.54, 8.27,	8.75, 8.67,			
**C**	7.88, 7.85	8.17, 8.05			
**D**					

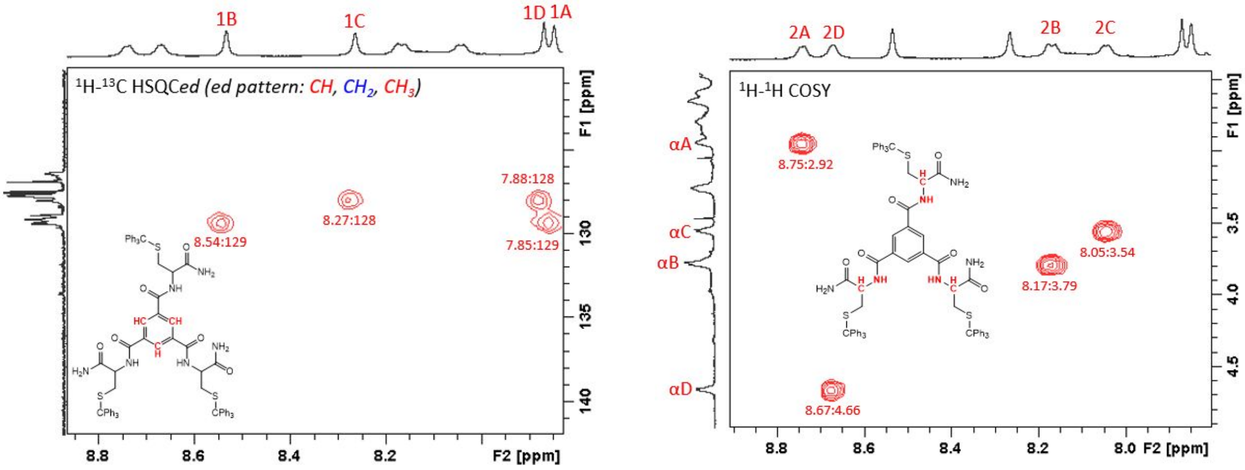

Figure S19. Parts of the ^{1}H-^{13}C HSQC NMR and COSY NMR (600 MHz TCE-d_2) spectra of L-**1**+L-**2** in 1:1 ratio.

2. Upon assignment of **peaks 2** in ^{1}H-^{1}H COSY experiment, the positions of four **α-peaks** have been also obtained (at 4.66, 3.79, 3.54, 2.92 ppm). Their localisation has been further confirmed in ^{1}H-^{13}C HSQCed experiment, which, thanks to the multiplicity edition, can distinguish $^{1}J_{C\text{-}H}$ CH (**α-peaks**) and $^{1}J_{C\text{-}H}$ CH_2 resonances (**β-peaks**).

	H^1	H^2	$H^α$	$H^β$	H^3
**A**					
**B**	8.54, 8.27,	8.75, 8.67,	4.66, 3.79,		
**C**	7.88, 7.85	8.17, 8.05	3.54, 2.92		
**D**					

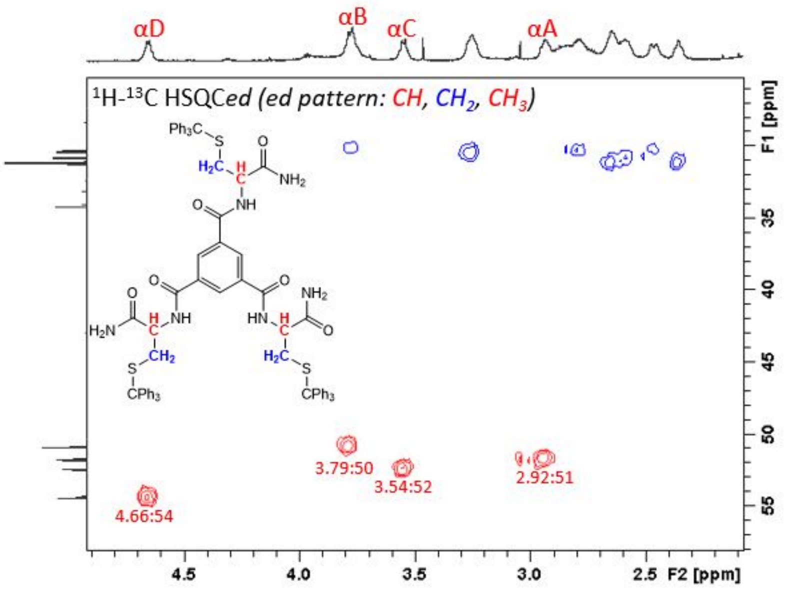

Figure S20. Part of the ^{1}H-^{13}C HSQC NMR (600 MHz TCE-d_2) spectrum of L-**1**+L-**2** in 1:1 ratio.

3. To match **peaks 1** and **peaks 2** to each other and assign them to the particular A-D groups ^{1}H-^{1}H ROESY technique was employed. In particular, four ROE effects were observed, as follows:

1A···2A 1B···2B 1C···2C 1D···2D.

This allowed for an unambiguous matching of **peaks 1** and **peaks 2**, and their assignment to individual groups A, B, C, D. Furthermore, since the **α-peaks** are corelated to **peaks 2** by $^{3}J_{H\text{-}H}$ COSY correlations, this also allowed for an indirect matching of the **α-peaks** to the A-D groups.

	H^1	H^2	H^α	H^β	H^3
**A**	7.85	8.75	2.92		
**B**	8.54	8.17	3.79		
**C**	8.27	8.05	3.54		
**D**	7.88	8.67	4.66		

Apart from ROEs, ROESY spectra often show exchange/TOCSY peaks. Those can be clearly distinguished from ROEs, based on the phasing. The spectrum revealed three of such exchange/TOCSY chain corelations, as follows:

1A···1C···1B **1B**···1A···1C **1C**···1A···1B

Notably, **1D group shows no correlations with A-C signals.** Therefore, it can be matched to the opposite enantiomer of **2**.

	H^1	H^2	H^α	H^β	H^3
**A**	7.85	8.75	2.92		
**B**	8.54	8.17	3.79		
**C**	8.27	8.05	3.54		
**D**	7.88	8.67	4.66		

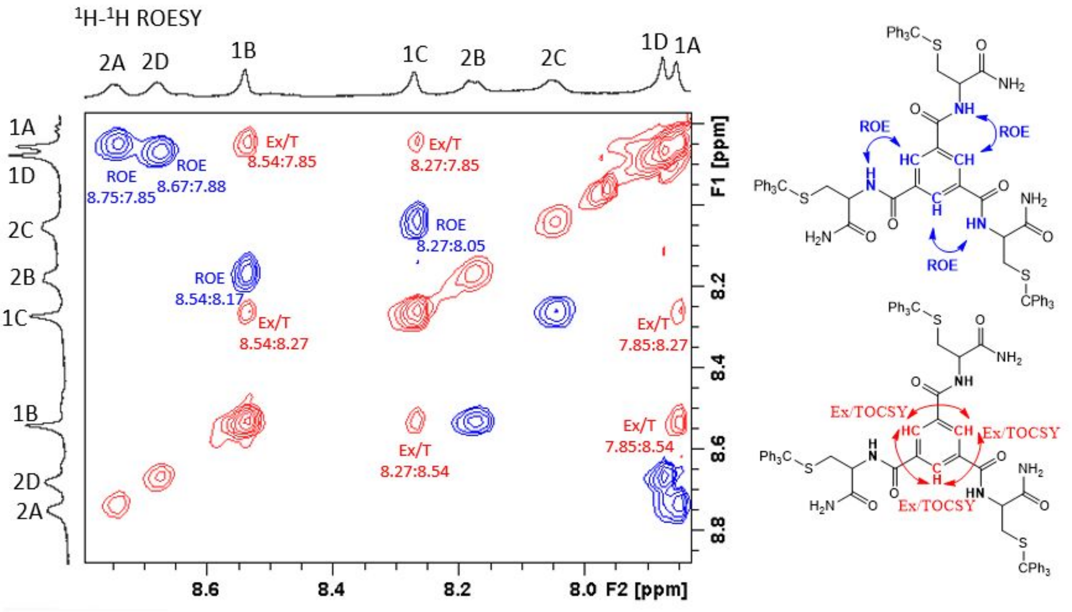

Figure S21. Part of the ROESY NMR (600 MHz TCE-d_2) spectrum of L-**1**+L-**2** in 1:1 ratio.

4. After assigning the **peaks 1, 2** and **α**, **β-peaks** can be unambiguously matched upon analysing $^3J_{H\text{-}H}(\boldsymbol{\alpha}-\boldsymbol{\beta})$ *and* $^2J_{H\text{-}H}(\boldsymbol{\beta}-\boldsymbol{\beta'})$ couplings in ^{1}H-^{1}H COSY spectrum.

	H^1	H^2	H^{α}	H^{β}	H^3
**A**	7.85	8.75	2.92	3.77<br>2.47	
**B**	8.54	8.17	3.79	3.25<br>2.59	
**C**	8.27	8.05	3.54	3.26<br>2.79	
**D**	7.88	8.67	4.66	2.64<br>2.35	

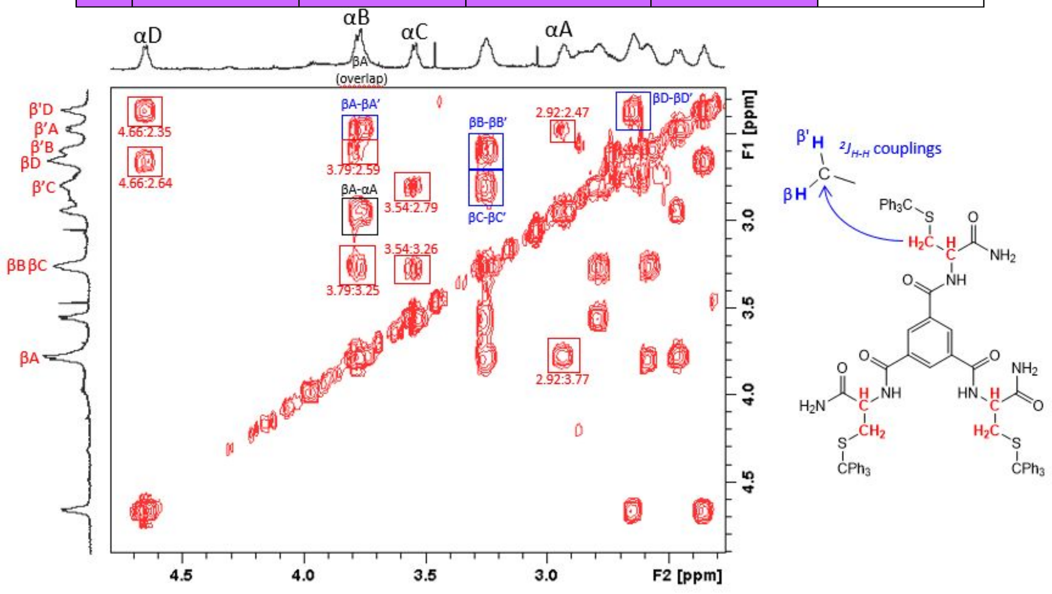

Figure S22. Part of the COSY NMR (600 MHz TCE-d_2) spectrum of L-**1**+L-**2** in 1:1 ratio.

5. The residual **peaks 3** (terminal NH_2 groups) show no characteristic corelations at the monomeric level, except of the geminal $^2J_{H\text{-}H.}$ The analysis of ^1H-^1H COSY spectrum indeed revealed four pairs of **peaks 3:3'**, but did not allow to match them to the A-D groups.

<table>
<tr><th></th><th>H¹</th><th>H²</th><th>H^α</th><th>H^β</th><th>H³</th></tr>
<tr><td>A</td><td>7.85</td><td>8.75</td><td>2.92</td><td>3.77
2.47</td><td rowspan="4">6.13:7.14
5.85:6.84
5.11:7.61
6.95:7.24</td></tr>
<tr><td>B</td><td>8.54</td><td>8.17</td><td>3.79</td><td>3.25
2.59</td></tr>
<tr><td>C</td><td>8.27</td><td>8.05</td><td>3.54</td><td>3.26
2.79</td></tr>
<tr><td>D</td><td>7.88</td><td>8.67</td><td>4.66</td><td>2.64
2.35</td></tr>
</table>

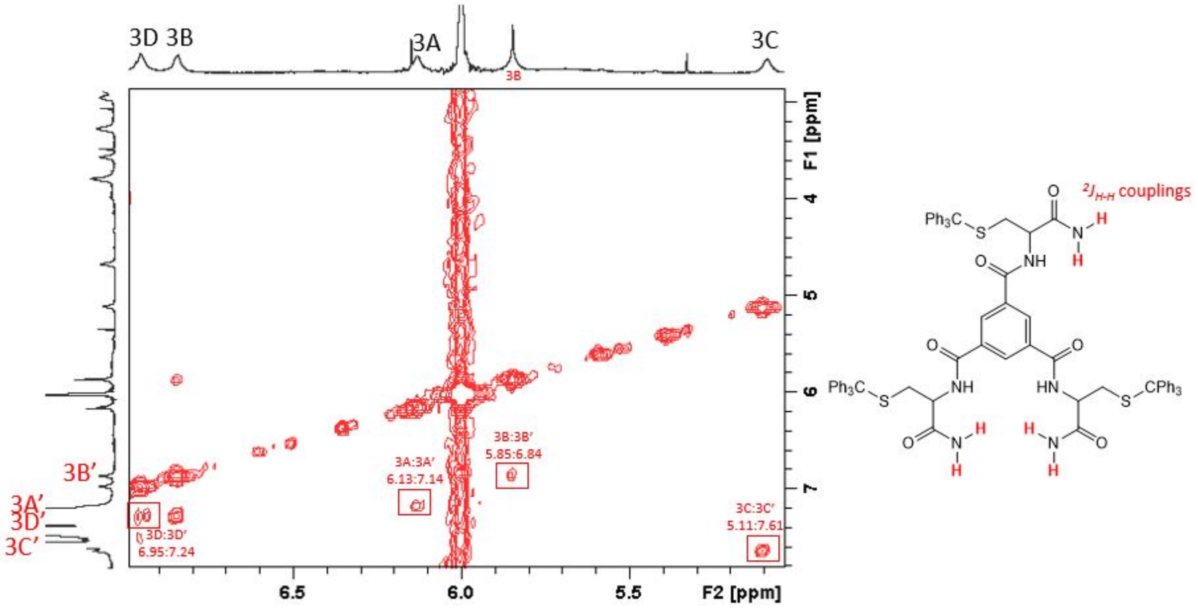

Figure S23. Part of the COSY NMR (600 MHz TCE-d_2) spectrum of L-**1**+L-**2** in 1:1 ratio.

6. However, upon analysing of the 3D model of LLLD-$\mathbf{2_4}$ one can observe, that **peaks 3** (not **3'**) are in close special proximity (<2.6 Å) with the corresponding **α-peaks**. ^1H-^1H ROESY analysis indeed revealed four **ROE** effects, as follows:

3A···αA 3B···αB 3C···αC 3D···αD.

This allowed for an unambiguous matching of **peaks 3** and their assignment to individual groups A, B, C, D. As noted earlier, **peaks 3**' are $^2J_{H\text{-}H}$ coupled to **peaks 3**, so their positions have been already determined.

	H^1	H^2	H^α	H^β	H^3
**A**	7.85	8.75	2.92	3.77<br>2.47	6.13<br>7.14
**B**	8.54	8.17	3.79	3.25<br>2.59	6.84<br>5.85
**C**	8.27	8.05	3.54	3.26<br>2.79	5.11<br>7.61
**D**	7.88	8.67	4.66	2.64<br>2.35	6:95<br>7.24

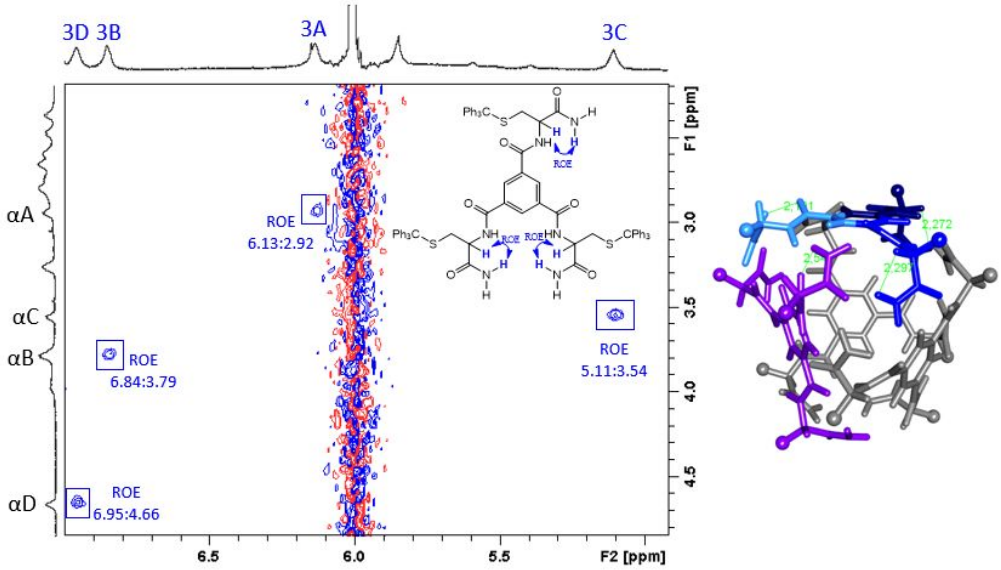

Figure S24. Part of the ROESY NMR (600 MHz TCE-d_2) spectrum of L-**1**+L-**2** in 1:1 ratio.

7. Further analysis of the ^{1}H-^{1}H ROESY spectrum and 3D model of LLLD-**2**$_4$ allowed for the positioning of individual groups A-D in 3D space, as well as to experimentally verify the symmetry of the model presented. Namely, proton **1A** should be in close proximity to **3D**, **1C** to **3B** while **1B** and **1D** shouldn't show any **ROE** effects. Indeed, both **ROE**s have been observed as follows:

1C···3B 1A···3D

	H^1	H^2	H^α	H^β	H^3
**A**	7.85	8.75	2.92	3.77<br>2.47	6.13<br>7.14
**B**	8.54	8.17	3.79	3.25<br>2.59	6.84<br>5.85
**C**	8.27	8.05	3.54	3.26<br>2.79	5.11<br>7.61
**D**	7.88	8.67	4.66	2.64<br>2.35	6:95<br>7.24

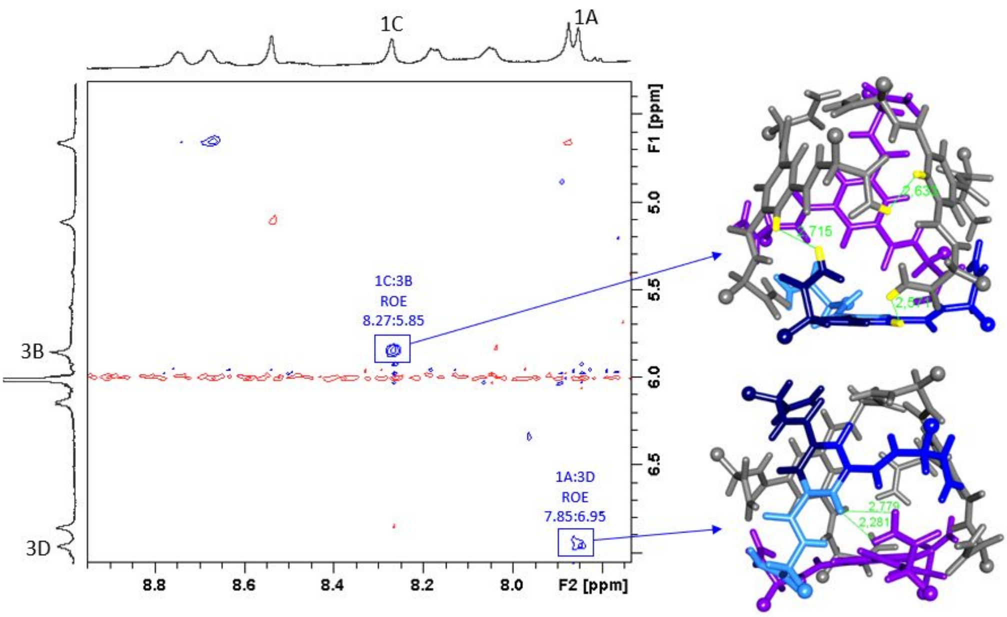

Figure S25. Part of the ROESY NMR (600 MHz TCE-d_2) spectrum of L-**1**+L-**2** in 1:1 ratio.

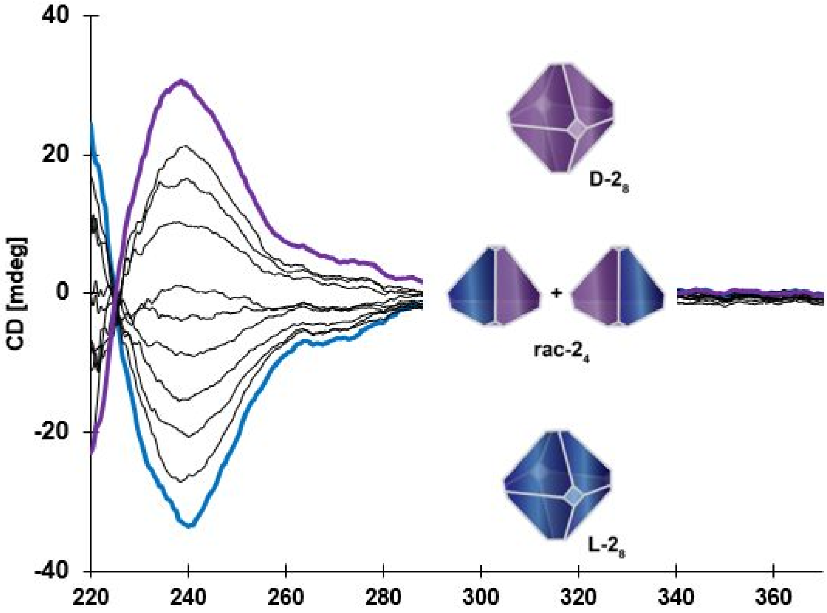

Figure S26. ECD spectra of the mixtures of L/D-**2** (10% molar increment) in DCE at 2.5 10^{-4} M.

*NOTE: In this experiment, two equimolar solutions of L-**2** and D-**2** in DCE were prepared and subsequently mixed at different ratios to afford the final of L/D-**2** mixtures. These measurements showed the expected linear dependence of the intensity of the Cotton effect on the concentration of individual enantiomers and a zeroing of the Cotton effect for the approx. 50:50 mixture, thus confirming the racemization and chiral purity of the starting materials. Albeit expected, this titration supplements the data obtained in ^{1}H NMR experiments, which - as an inherently achiral technique - cannot provide unambiguous information on the assembly between 2:6 and 6:2 ratio.*

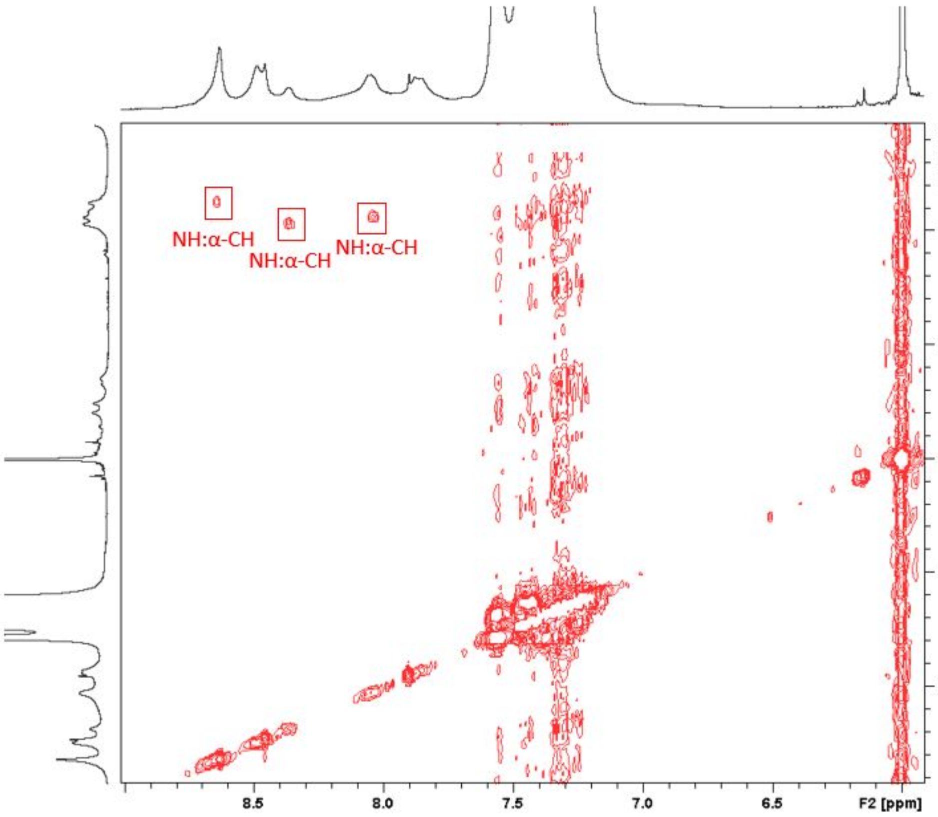

Figure S27. part of the COSY NMR (600 MHz TCE-d_2) spectrum of L-$\mathbf{2}_8$.

1.6 Fullerene encapsulation

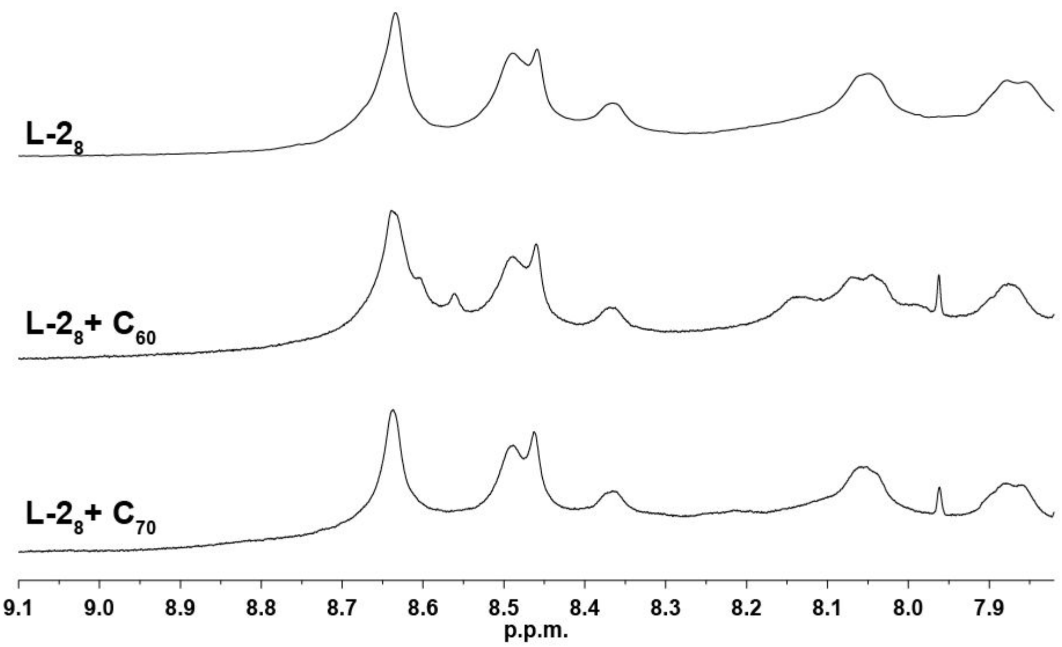

Figure S28. Part of the ^{1}H NMR spectra (600 MHz TCE-d_2) spectrum of L-**2**$_8$ without and with C_{60} and C_{70} fullerenes.

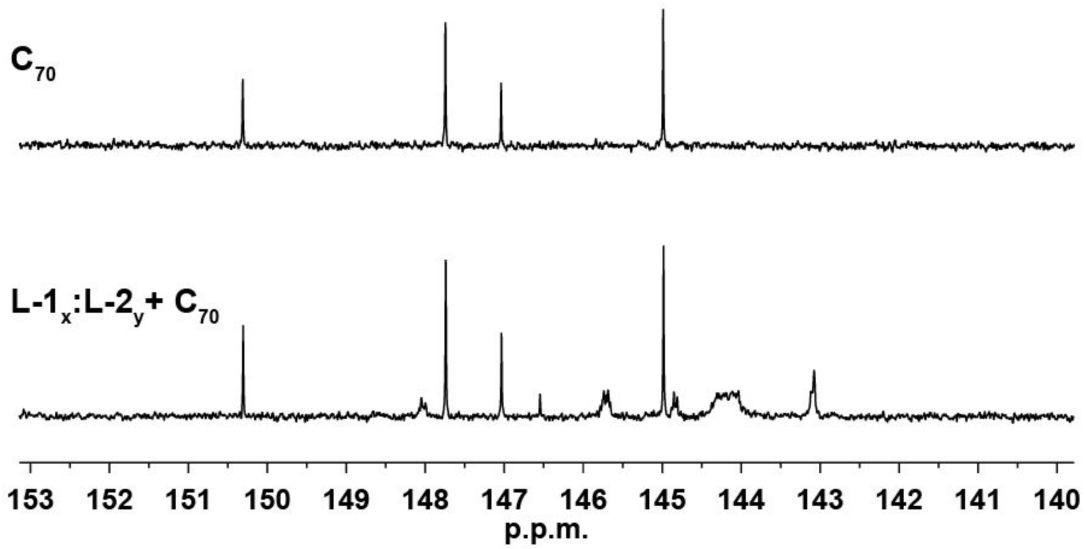

Figure S29. Part of the ^{13}C NMR spectra (151 MHz TCE-d_2) of C_{70} and L-**1**$_x$:L-**2**$_y$ + C_{70} mixture.

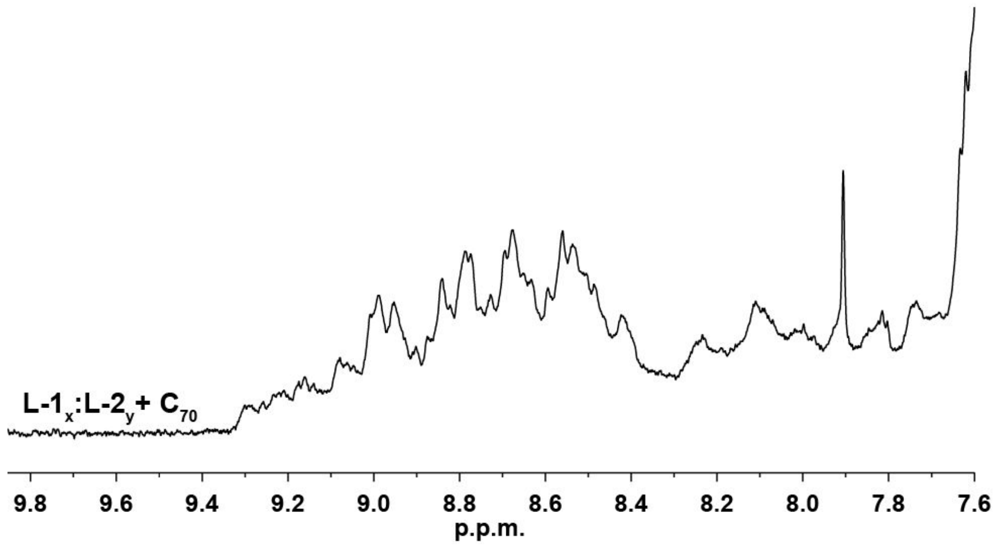

Figure S30. Part of the ^{1}H NMR spectrum (600 MHz TCE-d_2) L-**1**$_x$:L-**2**$_y$ + C_{70} mixture.

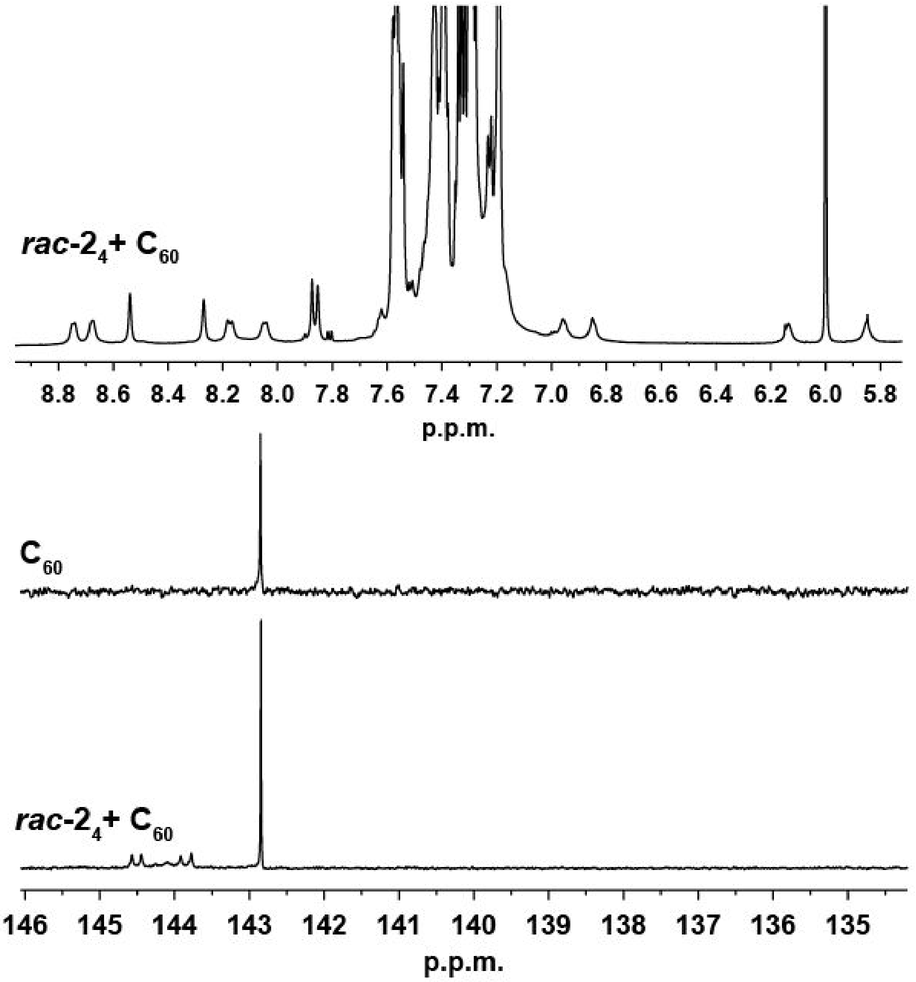

Figure S31. Part of the ^{1}H (600 MHz TCE-d_2) and ^{13}C NMR (151 MHz TCE-d_2) spectra of rac-**2**$_4$ with C_{60} fullerene.

2. Computational Procedures

The hydrogen bonded capsules and dimers, L-$\mathbf{1}_8$, L-$\mathbf{2}_8$, LLLL-$\mathbf{2}_4$, LLLD-$\mathbf{2}_4$, LLDD-$\mathbf{2}_4$ and L-$\mathbf{3}_2$, were constructed with the supramolecular toolkit (*stk*) software.[5] The configuration of the enantiopure building block monomer L-**1** was retrieved from the crystal structure of L-$\mathbf{1}_8$, which was subsequently used to derive the other building blocks by changing the -COOH functional group in the L-$\mathbf{1}_8$ monomer to -NH_2 for L-**2**, and -OH for L-**3** (full procedure outlined below). These monomers were then used to build the capsules L-$\mathbf{2}_8$,, LLLL-$\mathbf{2}_4$, LLLD-$\mathbf{2}_4$, and LLDD-$\mathbf{2}_4$ and dimer L-$\mathbf{3}_2$.

Approach for the generation of representative computational models:

To build the capsules, the monomers were first simplified by switching the *S*(Trityl)-cysteine arms to methyl groups. In doing so, the chirality of the monomer is preserved, but the computational cost of the calculation is decreased without losing any fragments of the molecule involved in the key self-assembly interactions. The monomers were then placed at the center of facets of the octahedral or tetrahedral polyhedra, such that each *R*-group was aligned towards the inside of the platonic solid (Figure S28). Each monomer was rotated sequentially from 0° to 120° with a step size of 5° about the vector between the centroid of the polyhedra and the centroid of the monomer to enumerate all potential capsule configurations. For each given capsule, this rotation method ensured a fine grid scan that would find configurations of the capsule that maximized the number of hydrogen bonds between the molecular components, as calculated with the ChimeraX software.[6] All structures that maximized the number of hydrogen bonds were then further geometry optimized to determine the energetic difference between different configurations and assemblies. Geometry optimizations were carried out using DFTB+ version 23.1, with the GFN2-xTB Hamiltonian included within the tblite library version 0.3.0.[7-8] Hydrogen bonded capsules were geometry optimized within DFTB+ with the approximate normal coordinate rational function optimizer and a Hessian matrix diagonalization tolerance of 10^{-2}. To obtain the final structures, atomic coordinates of the lowest energy structures were extracted for Density Functional Theory (DFT) calculations with the CP2K software package[9] using the PBE functional,[10] def2-TZVP basis set,[11] a grid size of 400 Ry and a relative cut-off value of 100 Ry. Non-bonded interactions were included using the Grimme-D3 dispersion correction.[12] While the conformational search was extensive, there remains the possibility that additional conformations were not fully sampled by our approach, however, we expect that the structures and relative energies of the conformations presented here are representative.

Validation of the computational approach:

To validate our methodology, a molecular representation from the crystal structure of L-$\mathbf{1}_8$ was overlaid with the computational model constructed with the above approach, as shown in Figure S29. The root

mean squared displacement (RMSD) values between experimental and computational structures of the hydrogen bonded capsules were calculated with Mercury software, with hydrogen atoms excluded from the RMSD calculations.[13] Our calculations do not include solvation effects and explored a fraction of configurational phase space that relied on the assumption that maximizing the number hydrogen bonds would lead to maximum capsule stability. But even with these simplifications, there was a good agreement between the X-ray diffraction structure and the generated computational model for L-$\mathbf{1}_8$, with an RMSD of 1.14 Å. This has promising implications for the computational structural generation of the other capsules where experimental structures were not available, such as for L-$\mathbf{2}_8$, as shown in Figure S30.

For the octahedral topology, the capsule monomer orientations of L-$\mathbf{2}_8$ were adapted from the L-$\mathbf{1}_8$ computational model. For the tetrahedral topology, our methodology produced 24^4 (331,776) possible structures. After discarding structures with atomic overlap (5% of structures) and without hydrogen bonds (0.5% of structures), we analyzed the configurations to find the structures with the highest number of hydrogen bonds, as determined by ChimeraX software. This analysis produced 5 LLLL-$\mathbf{2}_4$ configurations, 1 LLLD-$\mathbf{2}_4$ configuration, and 14 LLDD-$\mathbf{2}_4$ configurations with 9, 9, and 7 hydrogen bonds per capsule, respectively. These configurations were selected for further analysis to determine the energetic difference between the hydrogen bonded capsules. After GFN2-xTB geometry optimization, the maximum number of hydrogen bonds for each capsule increased from, 9 to 36 for LLLL-$\mathbf{2}_4$, 9 to 36 for LLLD-$\mathbf{2}_4$ and 7 to 28 for LLDD-$\mathbf{2}_4$.

The computational model for the L-$\mathbf{3}_2$ dimer is illustrated in Figure S31. The anticipated structure revealed an interplanar end-to-end distance of 3.15 Å between the alpha-carbon of benzyl groups of each of the monomeric building blocks, which would account for 12 hydrogen bonds between OH···O=C groups. The hydrogen bonds were measured to be in an expected range for OH···O=C groups, at around 2.6-3.0 Å between the donor and acceptor oxygen atoms.[12]

To test whether simplifying the S-Tr units to methyl groups affected the results, S-Tr units were reincorporated into the L-$\mathbf{2}_8$, LLLL-$\mathbf{2}_4$, LLLD-$\mathbf{2}_4$ and LLDD-$\mathbf{2}_4$ models using the Macromodel Suite Graphics User Interface and the "add fragments" option.[14] To account for steric effects, each S-Tr unit was reintroduced by performing sequential minimisations, optimising only the S-Tr units keeping the rest of the supramolecular structure fixed. Each arm of the monomer pointed towards a vertex of a tetrahedron, aligning with the positions of S-Tr neighbours in a tetrahedral topology, where three monomer arms met at each vertex as the closest point of contact for reintroducing S-Tr units. Therefore in each vertex, S-Tr unit were reattached to the structure in sets of three, and were relaxed with OPLS4 force field, with a maximum of 2,500 optimisation steps and a gradient convergence criterion of 0.05 kJ^{-1} $Å^{-1}$ using the Polak-Ribiere Conjugate Gradient minimisation algorithm.[15] When all S-Tr units were re-introduced to the assemblies, a final geometry optimisation was performed on the whole supramolecular assembly with the same OPLS4 force field settings. Finally, the DFT protocol as described above was applied to compare

relative energies for L-$\mathbf{2}_8$, LLLL-$\mathbf{2}_4$, LLLD-$\mathbf{2}_4$ and LLDD-**24**, as shown in Figure 6a. The reintroduction of bulky side chains showed negligible atomic position change to the central capsule, with root mean squared distance between simplified models with methyl groups, and S-Tr reintroduced complete structures for L-$\mathbf{2}_8$ (0.09 Å), LLLL-$\mathbf{2}_4$ (0.06 Å), LLLD-$\mathbf{2}_4$ (0.04 Å), and LLDD-$\mathbf{2}_4$(0.06 Å), respectively, as shown in Figure S33.

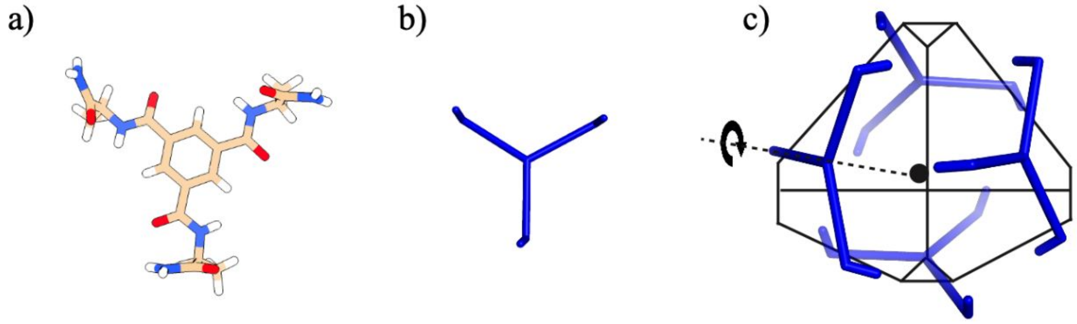

Figure S32. An example of the computational setup for modelling the arrangements of monomers in a tetrahedra capsule formation for L-**2**. a) Stick representation of the monomers where atoms are coloured as hydrogen (white), oxygen (red), carbon (yellow) and nitrogen (blue). b) Model simplified representation of the monomer shown in (a). c) Using the simplified representation; Placing the monomers around the platonic solid facets, in this case the tetrahedra, where each of the functional groups involved in hydrogen bonding are pointing into the tetrahedra to allow for maximum number of hydrogen bonds. The dashed line through the centroid of the tetrahedra and the molecule represent the axis of rotation explored when sampling different capsule configurations.

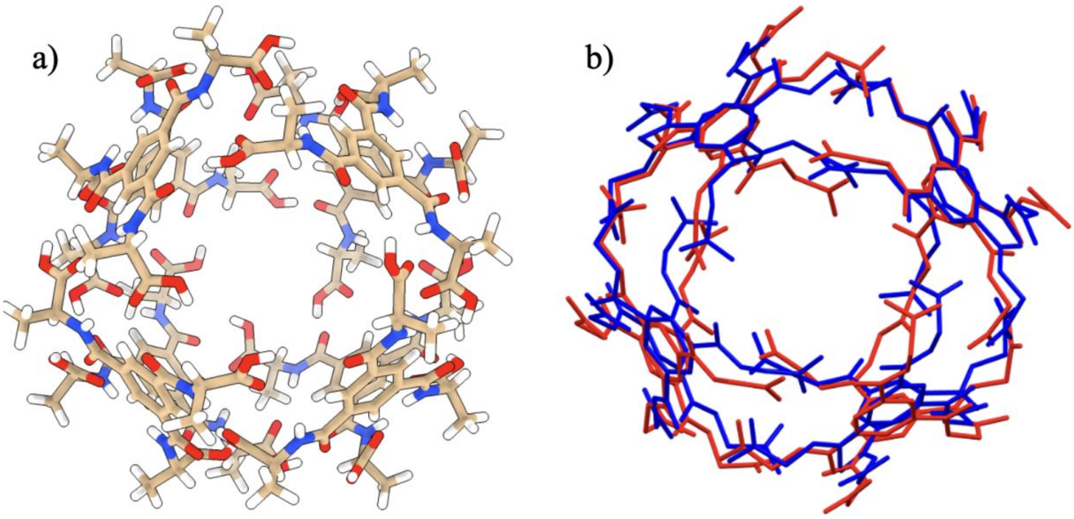

Figure S33. a) Stick representation for the computational model of L-$\mathbf{1}_8$; b) Overlay between computational model of L-$\mathbf{1}_8$ (blue) and the corresponding experimental crystal structure (red) (RMSD = 1.14 Å, hydrogens excluded). Atoms are colored as hydrogen (white), oxygen (red), carbon (yellow) and nitrogen (blue).

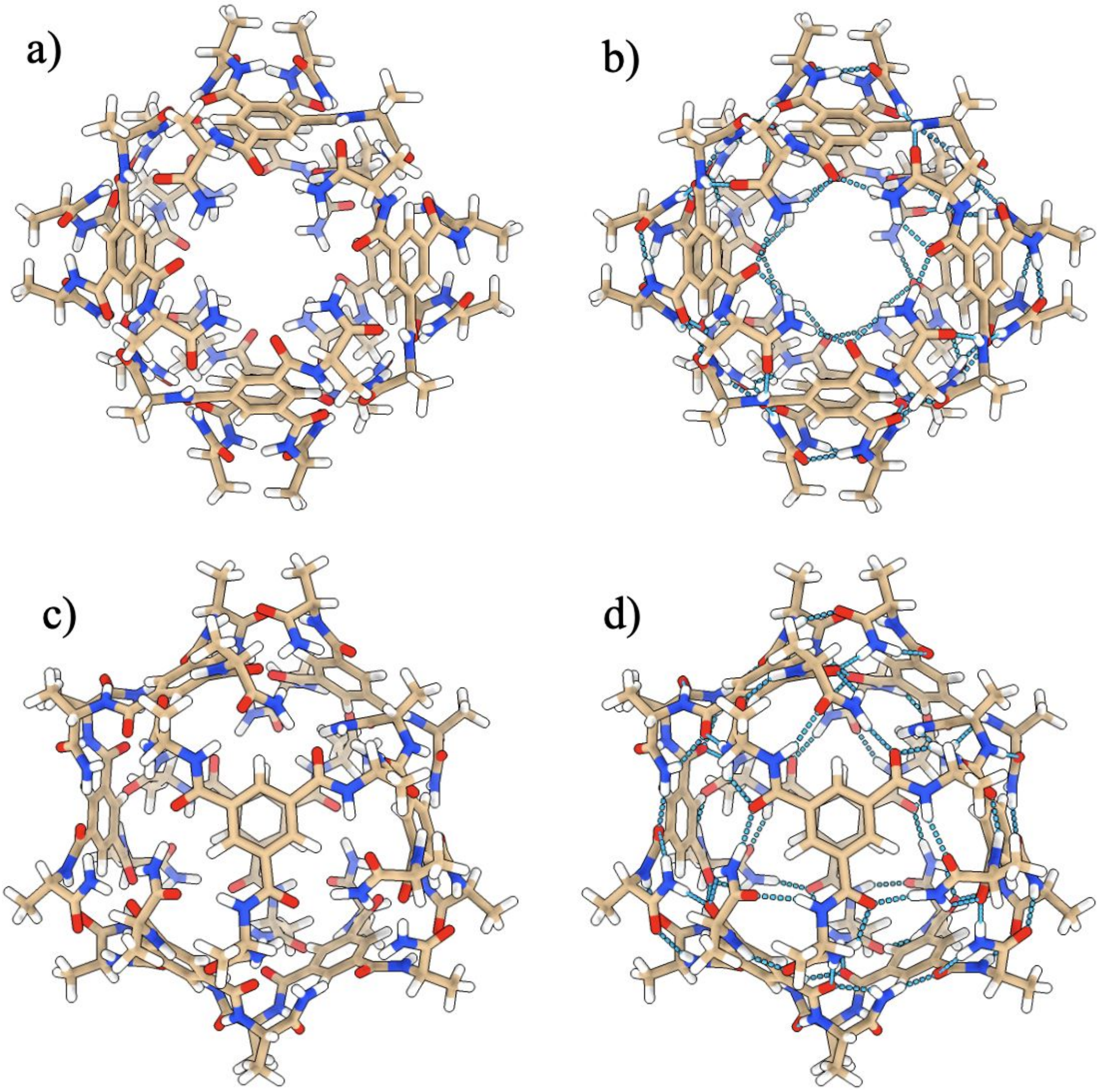

Figure S34. a) Stick representation for the computational model of L-$\mathbf{2}_8$ viewed down the a,b) *a*-axis c,d) *b*-axis, with b) and d) showing the hydrogen bonding network as represented by dashed cyan lines. Atoms are colored with respect to hydrogen (white), oxygen (red), carbon (yellow), nitrogen (blue).

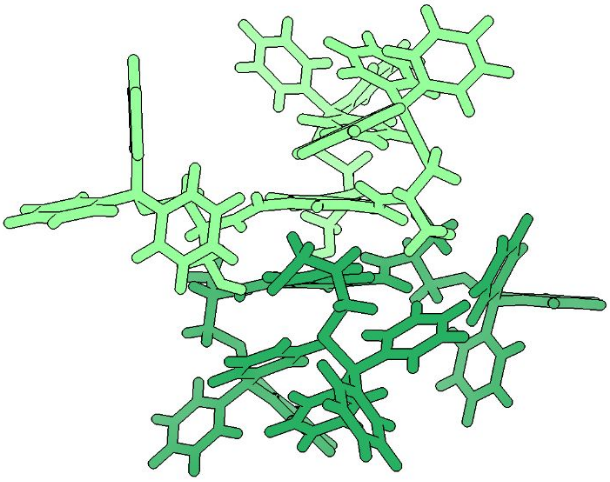

Figure S35. Computational models of the L-$\mathbf{3}_2$ dimer. Distinct monomers of the L-$\mathbf{3}_\mathbf{2}$ dimer are colored with dark green and light green.

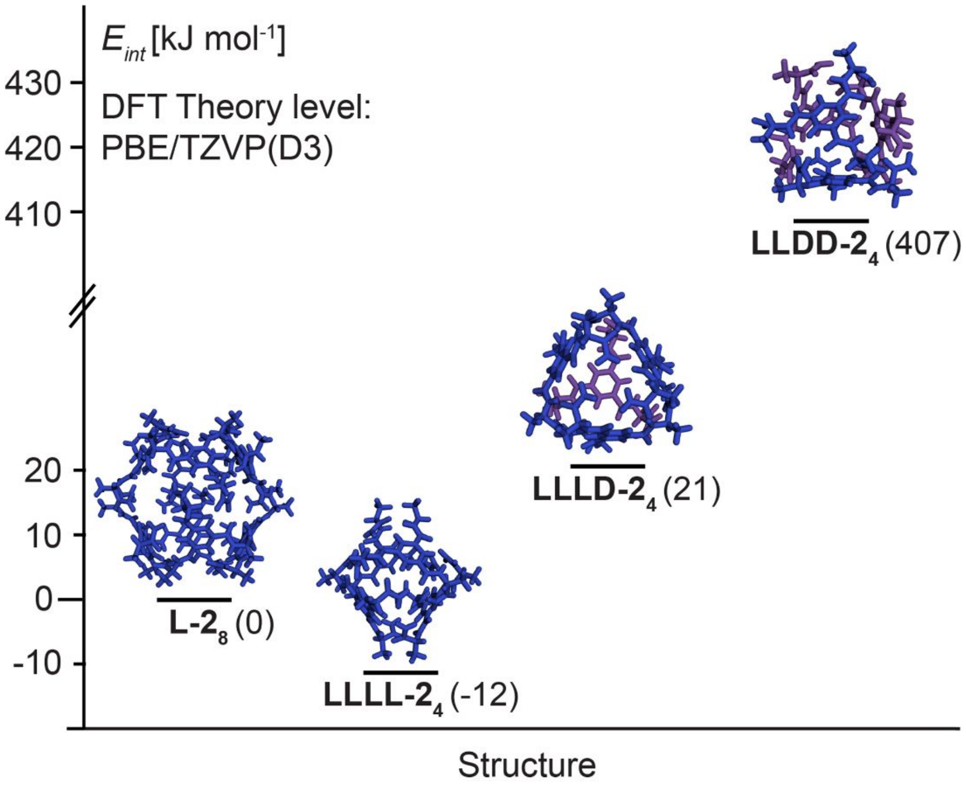

Figure S36. The lowest energy simulated conformation found for each hydrogen bonded assembly, before reintroduction of S-Trityl units: L-**2**$_8$, LLLL-**2**$_4$, LLLD-**2**$_4$ and LLDD-**2**$_4$, respectively.

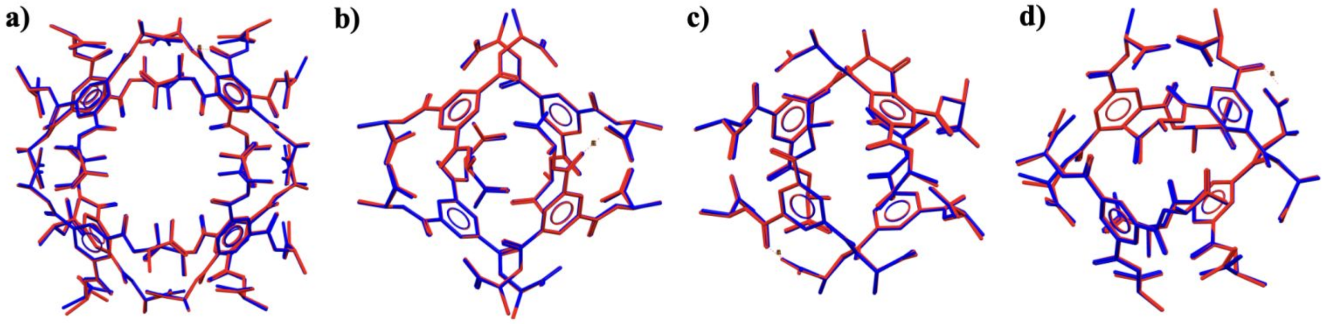

Figure S37. Overlay between simplified computational models of a) L-**2**$_8$, b) LLLL-**2**$_4$, c) LLLD-**2**$_4$ and d) LLDD-**2**$_4$ (blue) and the corresponding S-Tr reintroduced, DFT optimized computational models (red). RMSD = a) 0.09 Å, b) 0.06 Å, c) 0.04 Å, d) 0.06 Å, respectively for hydrogens excluded in L-**2**$_8$, LLLL-**2**$_4$, LLLD-**2**$_4$ and LLDD-**2**$_4$.

3. References

1. Brzechwa-Chodzyńska, A.; Markiewicz, G.; Cecot, P.; Harrowfield, J.; Stefankiewicz, A. R., Self-assembly of a fluorescent hydrogen-bonded capsule based on an amino-acid functionalised tetraphenylethylene. *Chem. Commun.* **2023,** *59* (41), 6247-6250.
2. Smulders, M. M. J.; Nieuwenhuizen, M. M. L.; de Greef, T. F. A.; van der Schoot, P.; Schenning, A. P. H. J.; Meijer, E. W., How to Distinguish Isodesmic from Cooperative Supramolecular Polymerisation. *Chem. Eur. J.* **2010,** *16* (1), 362-367.
3. Markiewicz, G.; Jenczak, A.; Kołodziejski, M.; Holstein, J. J.; Sanders, J. K. M.; Stefankiewicz, A. R., Selective C70 encapsulation by a robust octameric nanospheroid held together by 48 cooperative hydrogen bonds. *Nat. Commun.* **2017,** *8* (1), 15109.
4. J. I. Levin, J. M. C., Acetylenic sulfonamide thiol TACE inhibitors US 6313123 B1 Nov 06, 2001.
5. Turcani, L.; Berardo, E.; Jelfs, K. E., stk: A python toolkit for supramolecular assembly. *J. Comput. Chem.* **2018,** *39* (23), 1931-1942.
6. Pettersen, E. F.; Goddard, T. D.; Huang, C. C.; Meng, E. C.; Couch, G. S.; Croll, T. I.; Morris, J. H.; Ferrin, T. E., UCSF ChimeraX: Structure visualization for researchers, educators, and developers. *Prot. Sci.* **2021,** *30* (1), 70-82.
7. Bannwarth, C.; Ehlert, S.; Grimme, S., GFN2-xTB—An Accurate and Broadly Parametrized Self-Consistent Tight-Binding Quantum Chemical Method with Multipole Electrostatics and Density-Dependent Dispersion Contributions. *J. Chem. Theory Comput.* **2019,** *15* (3), 1652-1671.
8. Hourahine, B.; Aradi, B.; Blum, V.; Bonafé, F.; Buccheri, A.; Camacho, C.; Cevallos, C.; Deshaye, M. Y.; Dumitrică, T.; Dominguez, A.; Ehlert, S.; Elstner, M.; van der Heide, T.; Hermann, J.; Irle, S.; Kranz, J. J.; Köhler, C.; Kowalczyk, T.; Kubař, T.; Lee, I. S.; Lutsker, V.; Maurer, R. J.; Min, S. K.; Mitchell, I.; Negre, C.; Niehaus, T. A.; Niklasson, A. M. N.; Page, A. J.; Pecchia, A.; Penazzi, G.; Persson, M. P.; Řezáč, J.; Sánchez, C. G.; Sternberg, M.; Stöhr, M.; Stuckenberg, F.; Tkatchenko, A.; Yu, V. W. z.; Frauenheim, T., DFTB+, a software package for efficient approximate density functional theory based atomistic simulations. *J. Chem. Phys.* **2020,** *152* (12), 124101.
9. Kühne, T. D.; Iannuzzi, M.; Del Ben, M.; Rybkin, V. V.; Seewald, P.; Stein, F.; Laino, T.; Khaliullin, R. Z.; Schütt, O.; Schiffmann, F.; Golze, D.; Wilhelm, J.; Chulkov, S.; Bani-Hashemian, M. H.; Weber, V.; Borštnik, U.; Taillefumier, M.; Jakobovits, A. S.; Lazzaro, A.; Pabst, H.; Müller, T.; Schade, R.; Guidon, M.; Andermatt, S.; Holmberg, N.; Schenter, G. K.; Hehn, A.; Bussy, A.; Belleflamme, F.; Tabacchi, G.; Glöß, A.; Lass, M.; Bethune, I.; Mundy, C. J.; Plessl, C.; Watkins, M.; VandeVondele, J.; Krack, M.; Hutter, J., CP2K: An electronic structure and molecular dynamics software package - Quickstep: Efficient and accurate electronic structure calculations. *J. Chem. Phys.* **2020,** *152* (19), 194103.
10. Perdew, J. P.; Burke, K.; Ernzerhof, M., Generalized Gradient Approximation Made Simple. *Phys. Rev. Lett.* **1996,** *77* (18), 3865-3868.
11. VandeVondele, J.; Hutter, J., Gaussian basis sets for accurate calculations on molecular systems in gas and condensed phases. *J. Chem. Phys.* **2007,** *127* (11), 114105.
12. Grimme, S.; Antony, J.; Ehrlich, S.; Krieg, H., A consistent and accurate ab initio parametrization of density functional dispersion correction (DFT-D) for the 94 elements H-Pu. *J. Chem. Phys.* **2010,** *132* (15), 154104.
13. Macrae, C. F.; Sovago, I.; Cottrell, S. J.; Galek, P. T. A.; McCabe, P.; Pidcock, E.; Platings, M.; Shields, G. P.; Stevens, J. S.; Towler, M.; Wood, P. A., Mercury 4.0: from visualization to analysis, design and prediction. *J. Appl. Cryst.* **2020,** *53* (1), 226-235.
14. Lu, C.; Wu, C.; Ghoreishi, D.; Chen, W.; Wang, L.; Damm, W.; Ross, G. A.; Dahlgren, M. K.; Russell, E.; Von Bargen, C. D.; Abel, R.; Friesner, R. A.; Harder, E. D., OPLS4: Improving Force Field Accuracy on Challenging Regimes of Chemical Space. *J. Chem. Theory Comput.* **2021,** *17* (7), 4291-4300.
15. Polak, E.; Ribiere, G., Note sur la convergence de méthodes de directions conjuguées. *R.I.R.O.* **1969,** *3* (R1), 35-43.
